# Do relationships between leaf traits and fire behaviour of leaf litter beds persist in time?

**DOI:** 10.1371/journal.pone.0209780

**Published:** 2018-12-26

**Authors:** Zorica Kauf, Walter Damsohn, Andreas Fangmeier

**Affiliations:** Department of Plant Ecology and Ecotoxicology, Institute of Landscape and Plant Ecology, University of Hohenheim, Stuttgart, Germany; Centre National de la Recherche Scientifique, FRANCE

## Abstract

Wildland fires are a dominant disturbance on Earth. On the local scale, fire activity is also influenced by species-specific fire behaviour of leaf litter beds. Thus, researchers strive to identify plant functional traits governing fire behaviour. The currently accepted relationships between morphological characteristics of the individual particles, fuel bed structure and resulting fire behaviour have been established on freshly constructed leaf litter beds. To investigate to what degree these relationships are altered upon exposure of constructed leaf litter beds to outside weather conditions, a novel testing system was designed. It enables outdoor exposure of the constructed litter beds, their subsequent retrieval and fire behaviour testing without disturbing the sample structure. Two treatments were applied on seven monospecific leaf litters. “Fresh treatment” corresponded to the common practice of testing fire behaviour directly after fuel bed construction. In the “settled treatment” constructed fuel beds were exposed for 30 days to outside weather conditions before being tested. The “settled treatment” was designed to address physical changes in the fuel bed structure which occur due to repeated wetting of the fuel bed. Thus, to minimise the effects of decomposition and fragmentation, winter exposure was chosen. Within the “fresh treatment” previously established relationships between size, curl, bulk density and fire behaviour characteristics could be confirmed. In the “settled treatment” the majority of these relationships lost their significance. The “settled treatment” had significantly lower bulk density (BD), rate of spread, maximum flame height and maximum sand temperature at 1 cm depth; and significantly higher flaming duration and amount of unburned residues compared to the “fresh treatment”. Species with low initial BD were more affected by the treatment than species with high initial BD. The abrupt change in the fire behaviour of some leaf litter beds and the loss of numerous relationships between morphological characteristics of the individual particles and fire behaviour characteristics upon settled treatment indicate that fast occurring changes in the fuel bed structure should be taken into consideration if we are to understand the relationships between functional traits and local fire activity.

## Introduction

For more than 420 million years wildland fires have been an essential part of the Earth system [1]. Currently, fire annually burns roughly 3% of the Earth’s land surface [2], removing parts of the land biomass while emitting gases (predominantly CO_2_, CO and H_2_O), aerosols and heat, thus affecting energy and matter cycles, global climate and local weather conditions, as well as human social systems [3–6]. As a dominant disturbance fire has pronounced effects on evolutionary processes controlling ecosystem structure, distribution, productivity and biodiversity [7–12]. Approximately 40% of the biomes worldwide have been ecologically influenced by accompanying fire regime [13], with extensive alterations in the fire regime potentially leading to fundamental changes in the respective ecosystems [14–16]. Fire regime is defined by intensity, severity, frequency, seasonality, fuel consumption and fire spread patterns [8]. It is controlled by interactions between topography, soil, vegetation, weather/climate, atmosphere composition, ignition sources and human activities [1,2]. Due to complex interactions between the factors controlling fire regime, effects of the expected global change on fire activity [17] can trigger large scale biological and geophysical feedbacks [2]. Thus, understanding the role of fire in an ecosystem is essential, not only for choosing appropriate fire management practices, but also for predicting effects of global change on fire regimes as well as feedbacks of the altered fire regimes on the Earth System.

Plant species differ in their fire behaviour (e.g.,[18–20]), and changes in species composition can alter the fire regime (e.g.,[21,22]). To extrapolate the effects found at species level on the ecosystem level, relationships between plant traits and fire behaviour are extensively investigated (e.g.,[2,18,19,23–25]). Chemical composition, physical properties, quantity, size and shape, compactness and arrangement of a fuel are identified as characteristics affecting fire behaviour [26]. When examining the relationships between morphological plant characteristics and the resulting fire behaviour, leaf size is often identified as an important functional trait governing fire behaviour of the vegetation fuels (e.g.,[18,25,27–29]). Studies focusing on the leaf litter relate an increase in particle size to: higher flame height, higher fuel consumption, faster rates of spread, higher heat release rate, and shorter flame residence time [18,25,27,28,30]. The same effects are attributed to an increase in leaf curl, partially due to its positive relationship to the particle size [18]. As these relationships are found consistently across different laboratory testing methods (e.g.,[18,25,27,28]) and due to their similarities to field observations [24], they are currently considered valid [10,31] and represent a basis for establishing conceptual relationships between leaf traits and the fire behaviour of leaf litter beds [32–34].

Nevertheless, these well established relationships do not take into account litter bed dynamics. Leaf litter beds are continuously changing. Exposure to outside weather conditions results in the compaction of the leaf litter [35]. Decomposition results in a time dependent mass reduction, a reduction in particle density and changes in the chemical composition (e.g.,[36,37]). Fragmentation affects size and shape of the particles (e.g.,[38,39]), and abscission adds new material atop of the existing litter bed [40]. All these processes can be considered a part of the fuel bed dynamics. So far only few studies directly measured the effects of litter bed dynamics on its fire behaviour [35,41–44]. These studies do account for changes in chemical composition [44] and particle size [42]. Nevertheless, they do not account for in-stand observed alterations of the litter bed structure [35], the mass loss induced by decomposition, or effects of the addition of new material atop of the existing litter bed.

To enable addressing the alterations of the fuel bed structure and the mass loss induced by decomposition while examining the effects of leaf litter aging on its fire behaviour, we designed a construction which allows outside exposure of standardized samples, their retrieval and fire behaviour testing while preserving the fuel bed structure formed during the exposure. Here, the results of the first full scale experiment in which this newly developed methodology was applied are presented. The focus of this initial study was on the component of the fuel bed dynamics which operates on a short time scale after leaf abscission, namely, physical alterations in the fuel bed structure caused by repeated wetting of the fuel. The relationships between leaf area, leaf curl, bulk density, and selected fire behaviour characteristics as influenced by short term exposure to outside conditions were investigated. Constructed fuel beds (sensu [45]) were used. Results of a common approach of testing the fuel beds immediately after construction were compared to results obtained when constructed fuels were exposed outside for 30 days to winter weather conditions. We address the potential novelties of the herein presented methodology, in comparison to existing alternatives.

In accordance with our previous work [30] and work from other authors [10,31] attributing a single flammability value was not done. Furthermore, using the vegetation flammability definition introduced by [46] and expanded by [47] was avoided. This definition attributes four components to flammability (i.e. ignitibility, sustainability, combustibility, and consumability). Nevertheless, different authors attribute the same fire behaviour characteristics to different flammability components (e.g.,[31,48,49]). Thus, to avoid confusion, the suggestion given by [31] was followed and the metrics itself was addressed.

Our hypotheses were:

1. Samples constructed according to common approaches just prior to testing will confirm previously established relationships. For these samples increasing particle size and curl will result in: lower bulk density, higher flames, higher rate of spread, lower duration of flaming and higher fuel consumption.
2. The relationships between morphological characteristics of the individual particles (hereafter “particle characteristics”) and fire behaviour will not persist after 30 days of exposure to outside weather conditions.
3. Litter beds composed of larger particles with lower initial bulk density will be more affected by exposure to outside weather conditions than litter beds composed of smaller particles with higher initial bulk density. If this hypothesis holds true, between species differences in the bulk density would be lower in the settled compared to the fresh treatment. As bulk density exerts high control over fuel bed fire behaviour (e.g. [25]), smaller between species differences in bulk density would translate to lower between species differences in the fire behaviour characteristics.

Confirmation of our hypotheses would call for re-examination of the currently accepted relationships between leaf traits and fire behaviour of their litter. Furthermore, it would stress the importance of the fast occurring changes in the fuel bed structure in altering fire behaviour of the litter beds.

## Material and methods

### Sampling site and studied species

Leaf litter was collected in the Botanical Garden of the University of Hohenheim (Stuttgart, Germany) on 11. November 2016. Even though environmental conditions in the botanical garden may differ from those in the native environments of the sampled species, sampling in the botanical garden is plausible if general relationships are the focus of a study (e.g. [25,50]). The choice of species was based on diversity of their morphological characteristics and availability of the material. Studied species were: European ash (*Fraxinus excelsior* L.), Manchurian ash (*Fraxinus mandshurica* Rupr.), shingle oak (*Quercus imbicaria* Michx.), Lebanon oak (*Quercus libani* Olivier), pin oak (*Quercus palustris* Münchh.), spotted oak (*Quercus shumardii* Buckl.) and service tree (*Sorbus domestica* L.). Leaf litter is regularly gathered as a part of the garden maintenance. Thus, we can guarantee that only freshly fallen leaf litter was collected. Material was gathered during a rain event and was dried at 60°C for 96 hours before being stored in opened plastic boxes in a cool, dry room until further processing.

### Sample preparation and experimental setup

Sixteen samples per species were weighted (20 ± 0.1 g), with half of the samples randomly attributed to the “fresh” or the “settled” treatment. Within each treatment, 5 samples per species were used for testing fire behaviour and 3 for taking subsamples for morphological measurements. Samples assigned to the fresh treatment were prepared using the common approach of a sample being prepared just prior to the testing (e.g.,[18,25,28,51,52]). Fresh treatment samples were prepared by dropping a small amount of leaves at one time into the testing cage and consequently gently shaking the testing cage to ensure uniform distribution of the litter inside of the cage. The procedure was repeated until the whole sample was transferred to the testing cage.

Samples assigned to the settled treatment were exposed to outside weather conditions from 20. December 2016 to 19. January 2017. The initial sample preparation was the same as for the fresh treatment, however with the difference that the settled treatment samples were prepared in the exposure construction. This construction was composed of a 20 x 20 cm^2^ aluminium mesh bottom with a 2.5 cm high edge and a 13 cm high fabric enclosure. Each construction was fixed to the ground with four, 23 cm long, skewer tent pegs (S1 Appendix). The focus of this work lies on the physical alterations in the fuel bed structure caused by exposure to outside weather conditions, rather than biological decomposition. Thus winter was chosen as suitable period for exposure of the settled treatment samples, as low temperatures would minimize decomposition and fragmentation. Furthermore, as interception of the precipitation by tree canopies results in uneven wetting of the forest floor, a treeless environment was chosen for this, initial study. The experiment was established on a lawn as a random block design with 8 replicates, at the University of Hohenheim, Stuttgart, Germany. To prevent disturbance by animals and foreign material falling into the samples, a protection net covered the whole experiment. Temperature at the soil surface (Tinytag TGP-4017, Gemini Dataloggers Ltd., Chichester, UK), and air temperature and humidity at 40 cm height (Tinytag TGU-4500, Gemini Dataloggers Ltd., Chichester, UK) were logged with an hourly resolution from 23.12.2016 onwards (S2 Appendix).

Settled samples were retrieved with the whole exposure construction, left in the greenhouse for 24 hours to thaw, before drying at 60°C for 48 hours. Dry mass of all samples was determined. All samples were directly transferred to the fire behaviour testing facility after drying, where they were stored until testing. Samples were reweighted just before testing and moisture content was determined. Moisture content was low (mean = 5.12%) and showed only limited variation (S.E. = 0.158%), thus we do not present this data here.

### Measuring fire behaviour characteristics and fuel bed properties

To enable fire behaviour testing of the settled treatment samples without disturbance of the fuel bed structure, a special combustion chamber was developed (S3 Appendix). The stainless steel combustion chamber has an open front side providing air supply and an unobscured view of the burning samples. It is partially insulated with 2.5 cm thick vermiculite insulation board (V-1100 (700), Skamol, Nykøbing Mors, Denmark). A round opening in the roof (10 cm diameter) acts as an exhaust. A closable side door allows careful and precise handling of the sample. On the wall, opposite to the side door, 6 metal capillaries are present. Through these capillaries thermocouples are inserted. One pair of thermocouples records the temperature in the sand at 1 cm depth, the second pair records the temperature in the sample itself, and the third pair records air temperature above the sample (data not shown). An IR lamp (1000W, 1.4–1.7 μm, 90 kW/m^2^, SRSystems GmbH, Bruchköbel, Germany), aligned with the lower edge of the litter sample, acted as standardised linear ignition source. Ignition was piloted using a handheld spark generator. A digital camera (Power Shot SX280HS, Canon, Tokyo, Japan) was positioned in front of the combustion chamber, providing video records for all tests. The testing chamber was placed underneath a hood with its roof opening directly underneath the chimney opening of the hood. No air movement was detected at the top of the combustion chamber.

A 2 cm high frame, positioned inside the testing chamber, was filled with washed and size calibrated dry quartz sand at room temperature, creating a sand surface of 20.5 x 21 cm^2^. The sand surface was flattened and samples were carefully positioned on top of the sand. Positioning the samples on top of the sand, instead of compact fire-proof surface, enables measurements of the temperature increase of the substrate below the sample.

Samples were tested in the 14.5 cm high testing cage with an area of 21.0 x 21.5 cm^2^ (S4 Appendix). The testing cage is covered with a fine stainless steel mash and can be flipped open at the back side allowing settled treatment samples to be gently slid inside. The lower 2 cm of its front side are left uncovered to minimize overlapping of the testing cage mesh and the aluminium bottom of the exposure construction. Before testing of settled treatment samples, the fabric enclosure of the exposure construction was carefully removed, and samples were placed in the testing cage together with the aluminium bottom of the exposure construction. Thus, exposure induced changes in the structure of the settled treatment samples could be preserved. To ensure uniformity of the testing procedure, the same type of aluminium bottom was placed in the testing cage before samples assigned to the fresh treatment were constructed.

A stopwatch was started simultaneously with turning on the IR lamp. The spark generator was held in the smoke convection plume close to the fuel surface until flames appeared. Once flaming was initiated, the IR lamp was turned off. Times until: flame appearance, flame reaching back side of the fuel bed, flame extinguishment and last ember extinguishment were recorded. Unburned residues were weighted, transferred to Ni-cups of known weight and heated in the muffle oven for 8 hours at 550°C to determine their ash content.

Video and temperature records together with time measurements provide a wide basis for the extraction of numerous fire behaviour characteristics. To ensure comparability with previous works, we strived to include only commonly used and previously well-defined characteristics (e.g.,[18,25,45]): ignition delay (ID) as time required for the appearance of a flame once the ignition source is turned on, flaming duration (FD) as time for which flames are visible, smouldering duration (SD) as time between flame extinguishment and extinguishment of the last ember, maximum flame height (FH), rate of spread (RoS) as length of the fuel bed (20 cm) divided by the time required for the initiated flames to reach the back side of the fuel bed. FH was determined by frame by frame analysis of the video records. VideoPad video editor software (NCH Software, Greenwood Village, CO) was utilized to extract the frame of interest (S5 Appendix), and Image J [53] to measure the maximum flame height (S6 Appendix). Time was measured in seconds (s) with an accuracy of 0.01 s, FH in centimetre (cm) with an accuracy of 0.01 cm, and RoS is expressed in cm s^-1^.

Due to the distribution characteristics of our data set, we report “unconsumed” (UC) as the percentage of fuel remaining after burning (100%—% consumed), instead of the commonly reported consumed percentage of the fuel. Furthermore, as [31] correctly indicated that not separating ash from unburned remnants can lead to inaccurate assessment of the combustion completeness, we corrected UC for the ash content. UC, as presented here, was calculated as:

$$UC=\left(1-\frac{m_{i}{-m}_{uc}}{m_{i}-m_{ash}}\right)*100,$$

where *m*_*i*_ represents the initial sample mass, *m*_*uc*_ mass of the uncombusted rests and *m*_*ash*_ mass of the ash determined after combustion in the muffle oven. All masses were measured in grams (g) with an accuracy of 0.01g. Due to correction for the ash content, the reported UC value truly represents the proportion of the fuel which could potentially burn, but remained unconsumed. To account for potential differences in the effects of fire on the soil, we included the maximum sand temperature reached at 10 mm below the sand surface (ST) as an additional fire behaviour characteristic. This is of interest as effects of a fire on physical soil properties, soil seed bank, damage to the below ground plant parts and post-fire recovery of vegetation are directly related to the soil temperature [54–57]. Here, a highly standardised sand substrate was used as a soil replacement, thus the results do not correspond to field conditions. Nevertheless, they are comparable within the study. Furthermore, the frame could potentially be filled with any substrate of choice. ST was measured in °C with 0.01°C accuracy.

Due to logistical issues, samples assigned to fresh and settled treatments were tested on two different occasions. Fresh treatment samples were tested between 13. and 19. December 2016, settled treatment samples between 22. and 28. February 2017. Within the treatment, species were tested in blocks. Temperature and air humidity within the testing facility were logged (TGU-4500, Tinytag, Gemini Dataloggers Ltd., Chichester, UK) for the whole duration of the experiment. Average air temperature in the testing facilities was 21.2°C and air humidity 34.1%, with variation coefficients of 1.7% and 9.6%, respectively. No significant between treatment differences in temperature and air humidity were found (t-test P-value of 0.180 and 0.151, for temperature and relative humidity, respectively). To further exclude the possibility that our findings are the result of separate testing of fresh and settled treatments, we used surplus *Q*. *shumardii* material to prepare a sample according to fresh treatment procedure and test it on the same occasion as the settled treatment samples. All directly measured characteristics of this *Q*. *shumardii* sample fell within the range of values measured during fresh samples testing.

Sample height was measured at three random positions within the fuel bed just before testing. Sample volume was determined by multiplying sample area (area of the aluminium bottom, 40 cm^2^) with average sample height, and bulk density (BD) by dividing dry sample mass with sample volume. In our data set packing ratio and BD were highly related and exhibited similar relationships to area, curl (S7 Appendix) and fire behaviour characteristics (S8 Appendix). Thus, to stay concise, we focus on BD (expressed in kg m^-3^).

### Leaf litter particle characteristics

15 random leaf or leaflet particles were taken from 42 samples designated for morphological measurements (three random samples per each species and treatment combination). Both whole and fragmented particles were taken, as we were interested in the tendency of particles to break (data not presented here). All particles were photographed from above in their non-flattened and in their flattened state. ImageJ [53] was used to determine maximum width, maximum length, area (one sided surface of a particle) and perimeter of all, non-flattened and flattened, particles (in cm, with an accuracy of 0.01 cm). Additionally, the degree of curling (curl), defined as height above the flat surface [18], was measured with a ruler to the closest millimetre. Leaf thickness was measured with a digital calliper (in mm, with 0.01 mm accuracy).

Particles used for morphological measurements were either almost complete, or presented only a very small leaf/leaflet fragment (< 10% of the original leaf size) (S9 Appendix). Small fragments were relatively rare and attributed very little to the sample mass, but they had a disproportionally large effect on reducing average particle area. Thus we excluded small fragments from area calculation. This approach only slightly underestimates the standardised approach for measuring leaf area [58], making our results comparable to previous studies.

Despite the high number of characteristics determined, here we focus on the degree of curl of the particles in the fresh and the settled treatment, and on the area of the flattened particles from the fresh treatment. We do not address all the measured characteristics as they (i) showed low variability in our data (e.g., thickness, particle density), (ii) were highly correlated to area (e.g., perimeter, length, width, area in the settled treatment), or were (iii) not previously measured (non-flattened particles).

### Data analysis

The significance of the effect of species and treatment on the fire behaviour characteristics, BD and curl, was examined by Two-way ANOVA, with species and treatment as fixed factors. For area, One-way ANOVA with species as the fixed factor was used, as we only used the fresh treatment values. For curl and area, the respective subsample average value was used as the input data for ANOVA. For fire behaviour characteristics and BD, all 5 replicates per species x treatment were used as input data. In all cases, ANOVA was followed by pairwise contrasts with Tukey adjustment for multiple comparisons. Effect size was estimated as partial eta-squared [59].

As a novel method for testing fire behaviour was used, Principal Component Analysis (PCA) was applied on fire behaviour characteristics (ID, FD, FH, RoS, ST, UC, SD) to investigate if associations between them correspond to those previously established in similar studies. Furthermore, it was of interest to see how ST, a fire behaviour characteristic which is not commonly measured, is associated with commonly measured fire behaviour parameters. PCA was performed on scaled characteristics (mean = 0, standard deviation = 1). To account for full data variability and to achieve a satisfactory number of observations for the number of variables included [60], all measured values were included in the PCA (N = 70). If necessary, characteristics were transformed before scaling. The number of principal components (PC-s) to be retained was determined by the very simple structure (“vss”) function. The result of the PCA with four retained PC-s and the “varimax” solution rotation is reported.

To check the validity of our initial hypotheses three consecutive sets of regression analysis were conducted. The order of analysis followed the current understanding of the relationships between particle characteristics, fuel bed structure and fire behaviour. Following the premise that particle characteristics control fuel bed structure [28], the first set of bivariate regression analyses used area and curl as independent and BD as dependant variable. The second set of regression analyses followed the premise that particle characteristics[18,25,27,28] and fuel bed structure controls fire behaviour [61]. Here area, curl and BD were used to predict any of the fire behaviour characteristics. Both of these sets of regression analyses were performed separately on the fresh and the settled treatment, and on the whole data set. The third set of regression analyses was conducted to explore our third hypothesis (i.e. Litter beds composed of larger particles with lower initial bulk density will be more affected by exposure to outside weather conditions than litter beds composed of smaller particles with higher initial bulk density.) To test this hypothesis area, curl or initial BD (BD_f_, bulk density of the fresh treatment sample) were used as independent variables, and the percent difference between settled and fresh treatment mean species values (Δ) of the fire behaviour characteristics or BD as dependent variables. Δ was calculated as follows:

$$\Delta x=\frac{x_{s}-x_{f}}{x_{f}}*100,$$

where x is the characteristic of interest, x_f_ is the species mean value in the fresh treatment, and x_s_ is the species mean value in the settled treatment.

Since distribution characteristic can change depending on the used data subset, the significance of the linear, logarithmic and exponential relationship was tested. The relationship with the highest R^2^, respectively, is reported. We used only area of the fresh treatment samples, as this information is comparable to the information on which the current relationships between area, BD and fire behaviour characteristics are based. Regression analyses were performed with SPSS Statistics, version 24. All other analyses were performed using R [62].

## Results

### Significance of the effects of the species and treatment

**Fig 1:**
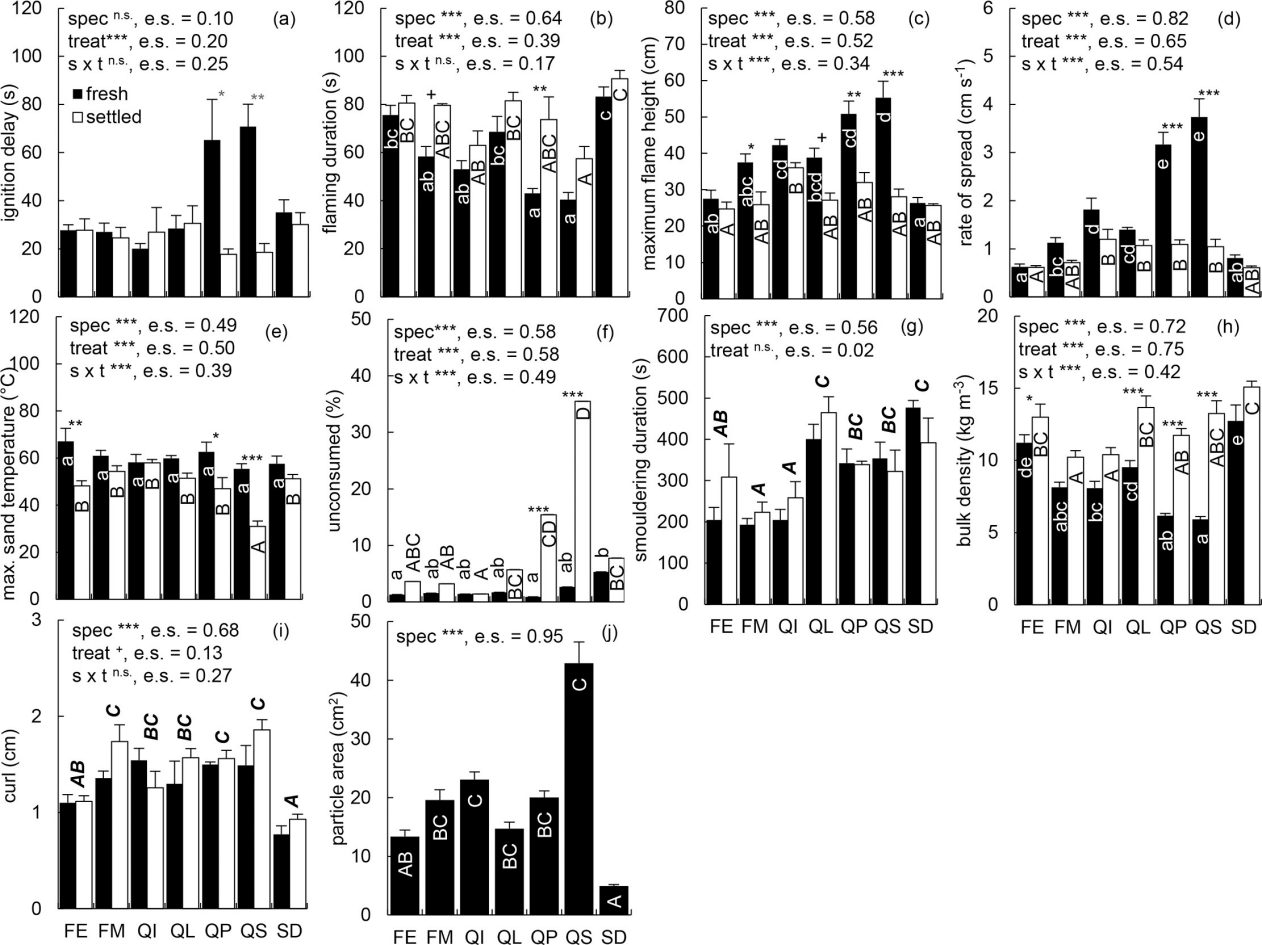
Means and standard errors with results of the ANOVA. **(a)** ignition delay (ID), **(b)** flaming duration (FD), **(c)** maximum flame height (FH), **(d)** rate of spread (RoS), **(e)** maximum sand temperature at 10 mm depth (ST), **(f)** unconsumed (UC), **(g)** smouldering duration (SD), **(h)** bulk density (BD), **(i)** curl and **(j)** particle area. Species are indicated as follows: *F*. *excelsior* (FE), *F*. *mandshurica* (FM), *Q*. *imbicaria* (QI), *Q*. *libani* (QL), *Q*. *palustris* (QP), *Q*. *shumardii* (QS), *S*. *domestica* (SD). Significance of species (spec), treatment (treat) and their interactions (s x t) was checked by means of Two -Way ANOVA for all characteristics except area, for which One-Way ANOVA with spec as fixed factor was applied. Effect size (e.s.), calculated as the partial eta-squared, is reported. ANOVA was followed by pairwise contrasts with Tukey’s adjustment for multiple comparisons. Different lowercase letters indicate statistically significant differences (P ≤ 0.05) within the fresh treatment, different capital letters indicate statistically significant differences within the settled treatment, and bold-italic capital letters are used to indicate significant between species differences when comparison was done only on the species level as treatment effects were not significant (SD and curl). For ID, ANOVA showed no significant species effect. Therefore, we do not report results of the within treatment between species pairwise contrasts for this characteristic. Significance of the fixed factor effects as indicated by ANOVA and significance of the within species treatment effects as determined by pairwise contrasts are indicated as: n.s. P > 0.10, + P ≤ 0.10, *P ≤ 0.05, **P ≤ 0.01 and ***P ≤ 0.001.

Results of the ANOVA (Fig 1.) show that ‘species’ as independent variable had no statistically significant (P > 0.05) effect on ID, and ‘treatment’ did not significantly affect SD and curl. For SD, curl, FD and ID interactions between species and treatment were not significant. All other tested effects were significant. Out of the six parameters on which both species and treatment had a significant effect, the settled treatment resulted in a decrease of FH, RoS and ST, whereas FD, UC and BD increased in the settled treatment. These trends were observed for the mean values of all species. Nevertheless, not all within species pairwise contrasts were significant. The settled treatment had a significant effect on all previously mentioned six characteristics measured in *Q*. *palustris*, and on all but one (FD) characteristics in *Q*. *shumardii*. *Q*. *palustris* and *Q*.*shumardii* were also the only species for which the settled treatment induced a significant decrease in ID. In contrast, within species comparison showed no significant differences between treatments, for any of the measured characteristics, in *Q*. *imbricaria* and *S*. *domestica*. Two characteristics (BD, ST) were significantly affected by ‘treatment’ within the species *F*. *excelsior*, one (BD) in *Q*. *libani* and one (FH) in *F*. *mandshurica*. In *Q*. *libani*, there was a trend towards a treatment effect on FH, and in *F*. *mandshurica* on FD (0.05 < P ≤ 0.10, respectively).

Differences of RoS and FH between species within one treatment showed a strong decrease in the range of measured values and in the number of significantly different groups in the settled compared to the fresh treatment. RoS identified five, and FH four, different groups within the fresh treatment, but they both differentiated only two groups within the settled treatment. For FH the range of measured values decreased from 50.22 cm in the fresh treatment to 25.62 cm in the settled treatment, and for RoS from 4.32 cm s^-1^ in the fresh treatment to 1.45 cm s^-1^ in the settled treatment. UC showed an opposite trend. Within the fresh treatment, UC of *S*. *domestica* was significantly higher in comparison to *F*. *excelsior* and *Q*. *palustris*, with no further significant pairwise contrasts. In the settled treatment four significantly different groups were identified based on UC values. Furthermore, the range of measured UC values increased from 7.82% in the fresh treatment to 41.96% in the settled treatment. BD showed a lower range and fewer significantly different groups within the settled compared to the fresh treatment, whereas ST exhibited a behaviour similar to UC. Nevertheless, for BD and ST levelling of the values in one treatment compared to the other was not as pronounced as for FH, RoS and UC.

### Relationships between fire behaviour characteristics

Results of the PCA (Table 1.) showed a clear separation of the fire behaviour characteristics. All characteristics had a loading higher than 0.85 on a single PC, and only one characteristic (ID) had a loading higher than 0.30 on an additional PC. PC1, which explained 36.5% of the total variation, was characterised by high positive loadings of RoS (0.936) and FH (0.867), and a high negative loading of FD (-0.887). This PC encompasses flaming related fire behaviour characteristics and indicates that faster spreading fires have higher flames, but shorter flame residence time. PC2, which explained 26.4% of the total variation, relates to effects of the combustion on the soil. It is characterised by a high negative loading of UC (-0.887) and a high positive loading of ST (0.951), indicating that a decrease in fuel consumption (increase in UC) results in lower soil temperatures. PC3 and PC4 are characterised by a high loading of one characteristic, SD and ID, respectively.

**Table 1:** Component matrix of fire behaviour characteristics based on the correlation matrix for the first four axes of a Principal Component Analysis (PCA).

t	PC1	PC2	PC3	PC4
explained variation	36.5%	26.4%	15.6%	14.1%
ln.ID	*0*,*315*	0,169	0,098	**0,929**
FD	**-0,887**	0,051	0,265	-0,119
SD	-0,065	-0,137	**0,975**	0,089
ln.RoS	**0,936**	0,131	0,072	0,221
ln.FH	**0,867**	0,295	0,030	0,172
ln.UC	-0,241	**-0,887**	0,226	-0,098
ST	0,028	**0,951**	0,012	0,110

Ignition delay (ID), flaming duration (FD), smouldering duration (SD), rate of spread (RoS), maximum flame height (FH), percentage of the fuel remaining upon combustion–unconsumed (UC), and maximum sand temperature at the 10 mm depth (ST) were used as input parameters. The PCA is based on standardised values (mean = 0, standard deviation = 1). ID, V, FH and UC were transformed (natural logarithm) before standardisation. The proportion of the total variance explained (% var) by the individual Principal Components (PC) is given.

### Predicting fuel bed structure and fire behaviour

**Fig 2:**
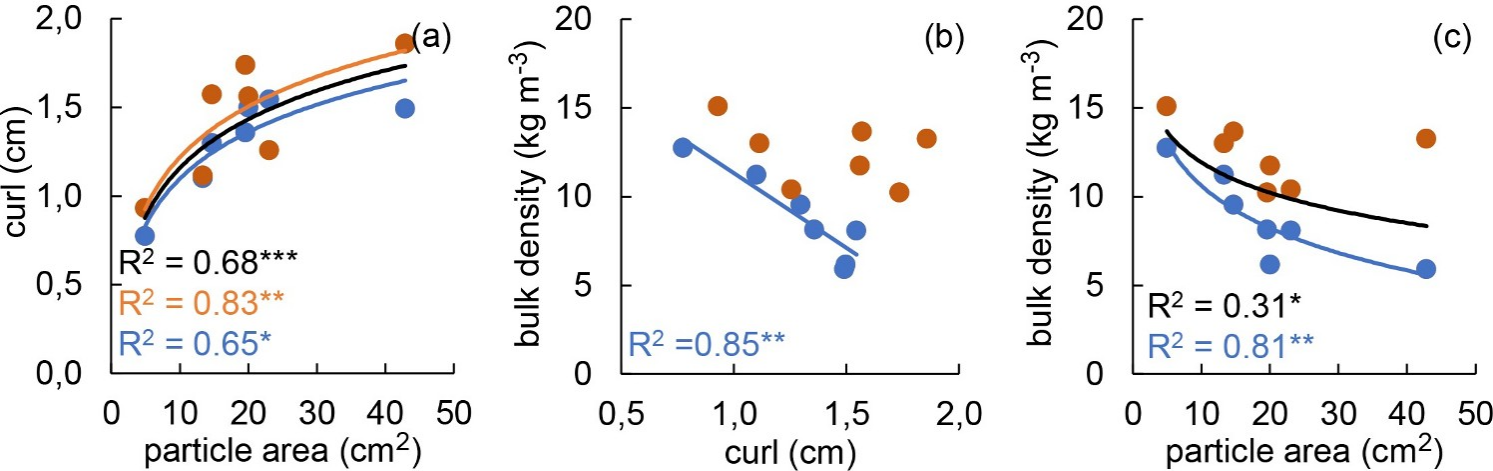
Pairwise regressions between particle area, curl and bulk density (BD). **(a)** particle area and curl, **(b)** curl and BD, **(c)** particle area and BD. The relationships were explored separately for the fresh (blue points, lines and R^2^ values) and the settled (orange points, lines and R^2^ values) treatment, as well as for the whole data set (black lines and R^2^ values). Regression lines and R^2^ values are presented only if significant (P ≤ 0.05). *P ≤ 0.05, **P ≤ 0.01 and ***P ≤ 0.001.

All three relationships between area, curl and BD were statistically significant (p ≤ 0.05) and strong (R^2^ > 0.6) in the fresh treatment (Fig 2A–2C). Between area and curl there were consistent positive relationships regardless of the data set used (fresh, settled, or the whole data set) (Fig 2A). However, the negative relationship between either of these parameters (i.e. area and curl) and BD lost its significance in the settled treatment (Fig 2B and 2C). The relationships between area and BD were significant for the whole data set (Fig 2C), nevertheless with a lower R^2^ (0.31) compared to the fresh treatment (0.81).

**Fig 3:**
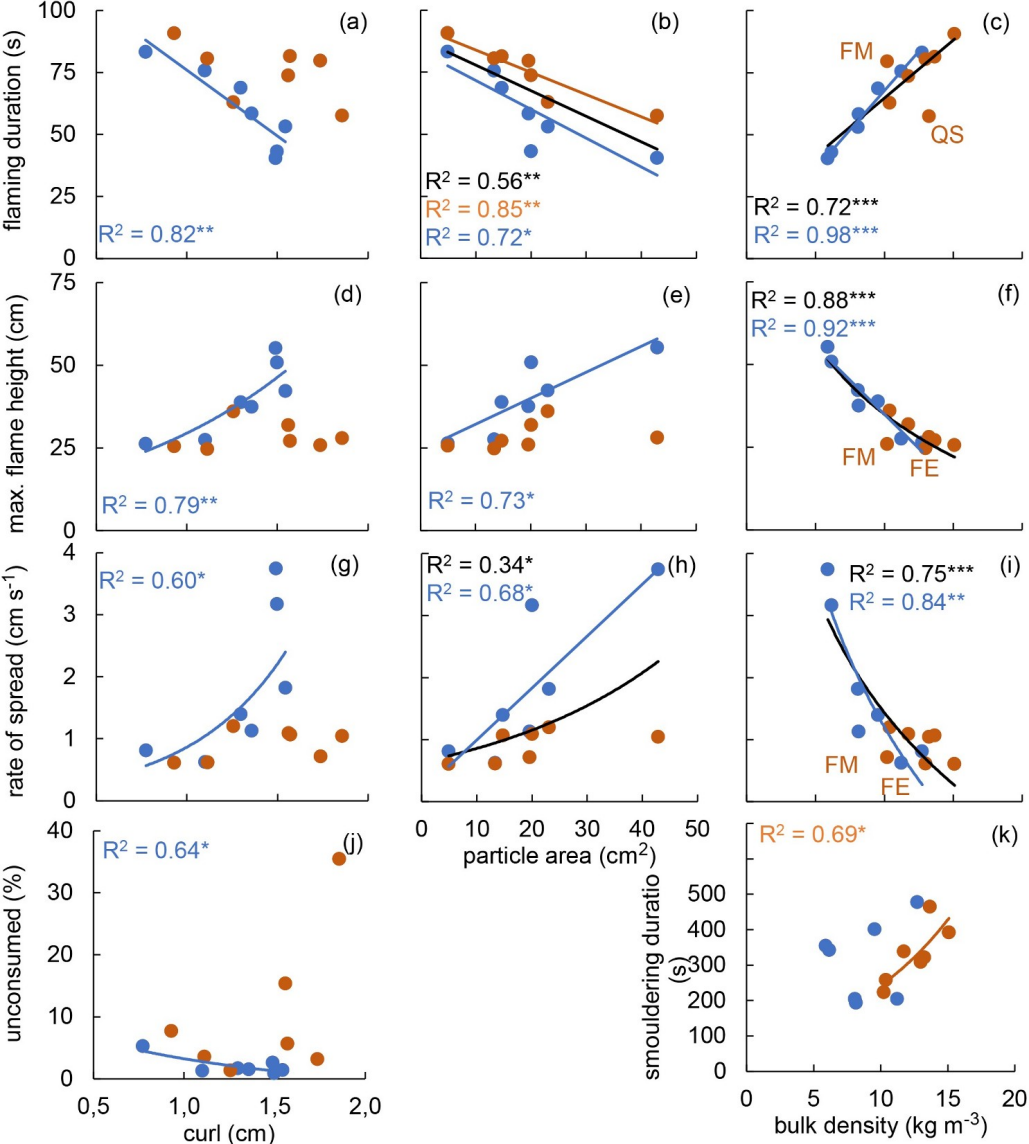
Pairwise regressions with particle area, curl and bulk density (BD) as independent and fire behaviour as dependent variables. **(a)** curl and flaming duration (FD), **(b)** particle area and FD, **(c)** bulk density (BD) and FD, **(d)** curl and maximum flame height (FH), **(e)** particle area and FH, **(f)** BD and FH, **(g)** curl and rate of spread (RoS), **(h)** particle area and RoS, **(i)** BD and RoS, **(j)** curl and percentage of the fuel remaining upon combustion–unconsumed (UC), **(k)** BD and smouldering duration. Analyses were performed for all 21 parameter pairs (curl, particle area or BD as independent and any of the seven fire behaviour parameters as dependent variables). For each pair, the relationship was explored separately for the fresh (blue points, lines and R^2^ values) and the settled (orange points, lines and R^2^ values) treatment, as well as for the whole data set (black lines and R^2^ values). Only pairwise relationships that were significant (P ≤ 0.05) for at least one level of comparisons (i.e. fresh, settled or whole data set) and had an R^2^ > 0.3 are presented here. Significance levels: *P ≤ 0.05, **P ≤ 0.01 and ***P ≤ 0.001.

When employing area, curl or BD as independent variables and fire behaviour parameters as dependent variables (Fig 3.), all three independent variables (curl, area and BD) exhibited a strong and significant relationship with flaming related parameters (FD, FH and RoS) within the fresh treatment. For the given relationships, an increase in either curl or area resulted in an increase of FH and RoS, and a decrease of FD. In contrast, an increase in BD resulted in a decrease of FH and RoS, and an increase of FD. Similar to the relationship between curl and BD (Fig 2B), all relationships between curl and fire behaviour parameters lost their significance in the settled treatment (Fig 3A, 3D, 3G and 3J). The same happened to the relationships between area and FH (Fig 3E), and area and RoS (Fig 3H), which were non-significant for the settled treatment. The relationship between area and FD (Fig 3B) was significant in both treatments, as well as for the whole data set. The relationship between area and RoS was also significant for the whole data set (Fig 3H), but with lower R^2^ (0.34) than in the fresh treatment (0.68). UC and SD were fire behaviour characteristics which exhibited only one significant relationship. UC was negatively related to curl in the fresh treatment (Fig 3J), and SD was positively related to BD in the settled treatment (Fig 3K).

All relationships between BD and fire behaviour parameters which were significant for the fresh treatment (i.e. BD-FD, BD-FH, BD-RoS) were also significant for the whole data set. Furthermore, within the fresh treatment, they were stronger (higher R^2^) and had a higher statistical significance (lower P-value) than the relationships between fire behaviour parameters and either area or curl. The lack of significant relationships between BD and fire behaviour parameters in the settled treatment was always driven by two data points, whereas the remaining 5 data points were almost perfectly aligned (e.g., R^2^ > 0.90).

**Fig 4:**
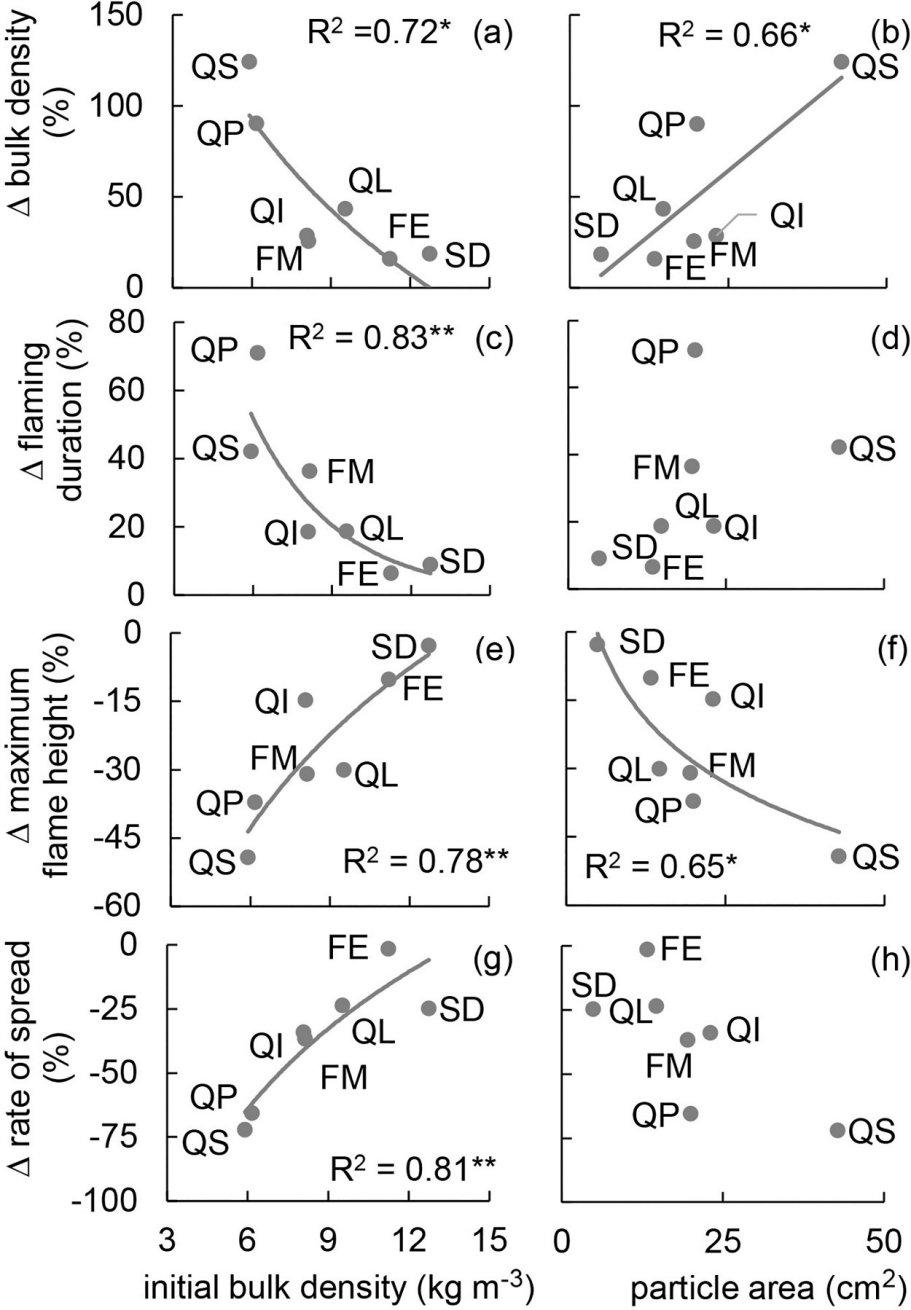
Percent difference (Δ) of fire behaviour parameters and bulk density (BD) as influenced by initial bulk density (BD_f_) or particle area. **(a)** BD_f_ and ΔBD, **(b)** particle area and ΔBD, **(c)** BD_f_ and Δ flaming duration (FD), **(d)** particle area and ΔFD, **(e)** BD_f_ and Δ maximum flame height (FH), **(f)** particle area and ΔFH, **(g)** BD_f_ and Δ rate of spread (RoS), **(h)** particle area and ΔRoS. Species mean values are presented and species are indicated next to the corresponding data point: *F*. *excelsior* (FE), *F*. *mandshurica* (FM), *Q*. *imbicaria* (QI), *Q*. *libani* (QL), *Q*. *palustris* (QP), *Q*. *shumardii* (QS), *S*. *domestica* (SD). Analyses were performed for all 24 parameter pairs (curl, area or BD as independent variables and Δ of the seven fire behaviour parameters and BD as dependent variables). Results are presented if Δ for the given fire behaviour parameter exhibited at least one significant (P ≤ 0.05) relationship. Relationships between curl and Δ are not shown as none of the relationships was significant. *P ≤ 0.05, **P ≤ 0.01 and ***P ≤ 0.001.

All Δ values for BD and FD were positive, indicating that they were higher in the settled than in the fresh treatment, whereas only negative Δ values for FH and RoS demonstrate that they were lower in the settled compared to the fresh treatment for all tested species (Fig 4). These findings are in accordance with the data shown in Fig 1 (Fig 1H, 1B, 1C and 1D). BD_f_ showed a significant positive relationship with ΔBD and ΔFD (Fig 4A and 4C), and a significant negative relationship with ΔFH and ΔRoS (Fig 4E and 4G). Nevertheless, for all four parameters, an increase in BD_f_ resulted in a decrease of the absolute Δ values, suggesting that with increasing BD_f_ the effects of the settled treatment on BD, FD, FH and RoS decreased. Area was significantly related to ΔBD and ΔFH (Fig 4B and 4F). The absolute Δ values of BD and FH increased with increasing area, thus indicating that the settled treatment exerted a larger effect on these parameters with increasing area.

None of the relationships using curl of the fresh treatment as predictor of the Δ values was significant. The same holds true for the relationships with ΔID, ΔSD, ΔUC or ΔST as dependent variable, regardless which parameter was used as independent variable. For more information on area and curl refer to S10 Appendix, and for information on the measured values of fuel bed structure characteristics and fire behaviour characteristics please refer to S11 Appendix.

## Discussion

### Comparison of relationships found in the fresh *vs*. settled treatment

The results obtained from the fresh treatment confirm our first hypothesis. There is consistency of the relationships found in our study in the fresh treatment with previous studies that applied a similar manner of sample construction. This indicates that the methodology applied here is capable of capturing relationships between particle characteristics, fuel bed properties and fire behaviour of the leaf litter beds. Consequently, the lack of significance for some of the relationships within the settled treatment cannot be attributed to an insensitivity of the testing method. The relationships we found within the fresh treatment coincide with the findings of [27], who reported a significantly negative relationship between leaf area and litter density (equivalent of BD), positive relationships between area and spread rate (equivalent of RoS), and area and FH, as well as a negative relationship between area and sustainability (equivalent of FD). While examining fire behaviour of Californian oaks [18], leaf curl measurements were included and showed that species with small, thick leaves and low curl burned with lower FH, had longer FD and low fuel consumption. In contrast, species with large, thin leaves and high curl had higher FH, shorter FD and substantial fuel consumption. Furthermore, there is also a relationship between leaf margin (lobed, entire) and fire behaviour. Lobed leaves form leaf litter beds which burn with greater fire intensity than leaf litter beds composed of leaves with entire margin [18,63]. These observations coincide with our fresh treatment results, as the two species with lobed leaves (*Q*. *palustris* and *Q*. *shumardii*) where the ones with the highest FH, highest RoS and the shortest FD. In agreement with [18], large, curled leaves in our study also tend to be lobed, thus it is difficult to separate effects of individual traits. Further laboratory [25,50] and field [24] studies also identified particle size as a first order trait determining the fire behaviour of leaf litter. To explain these results, all mentioned studies resort to experimental work of [28]. These authors showed that larger particles form leaf litter beds with lower packing ratio (and BD) resulting in higher fuel bed aeration, better heat penetration into the fuel bed and higher reaction velocities. This explanation complies with the theoretical background provided by [61]. Due to the high level of consistency between different studies, the postulated effects of particle size and curl on the fire behaviour of leaf litter beds are considered valid [2,10,31] and represent the basis for establishing conceptual relationships between plant traits and fire behaviour [32,34]. Unfortunately, previous studies and proposed conceptual frameworks do not take into account changes in the fuel bed structure.

We made the same observation as [35] namely, hardwood leaf litter tends to quickly compact (“settle”) after abscission when exposed to local weather conditions. Such effects have not been measured in previous studies. Rather, previous studies have tested leaf litter beds constructed using dry particles. The only exception from this have been experiments addressing the effects of moisture content on fire behaviour of litter beds (e.g., [52,64,65]). Furthermore, leaf litter beds are commonly tested as soon as possible after they have been prepared. Depending on their size and shape, dry leaf litter particles tend to form litter beds with specific maximum bulk density, which cannot be increased without breaking the particles [28,34,52,66].

**Fig 5:**
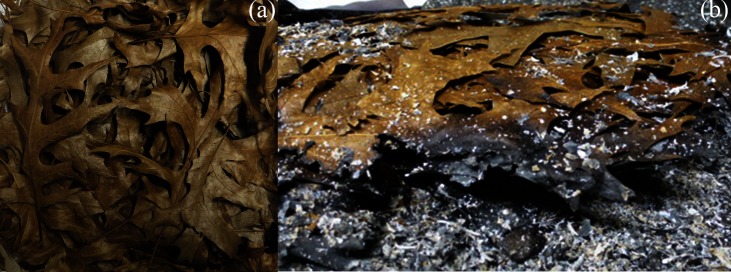
Photographs of the settled *Q*. *shumardii* sample. **(a)** view of the upper surface of a sample from *Q*. *shumardii* from the settled treatment prior to fire behaviour testing, **(b)** turned over unburned rests showing the lower side of this sample.

An increase in moisture content tends to decrease the particle rigidity and breakability, thus decreasing the particles’ resistance to compression. In this study and in our previous work [30,42] we used this property to flatten leaf litter particles for surface measurements. Furthermore, as particles absorb water their fresh mass inevitably increases and they exert higher pressure on the particles below them. Higher pressure together with lower resistance to compression can lead to compaction of the leaf litter beds, flattening of the particles within the fuel bed complex and an increase in BD. The strong and highly significant effect of treatment on BD (Fig 1H) indicates that sample compaction after 30 days of winter exposure to outside weather conditions (i.e. the settled treatment) is substantial and not reversed after drying. Although we did not measure the fuel bed depth dependent BD in the settled treatment, the presence of partially curled particles on top of the settled fuel (Fig 5A) and of flattened particles within the fuel bed (Fig 5B) strongly indicates the formation of a BD gradient within the fuel bed.

Similar to particles on top of the settled fuel bed, individual particles separated from the fuel bed complex may regain their initial curl after drying, as indicated by the lack of a significant effect of the treatment on the curl (Fig 1I). After the settling treatment large particles can exist in a flattened and densely packed state in the fuel bed (Fig 5B), and in a highly curled state if separated from the fuel bed. Thus, particle characteristics become unreliable predictors of fuel bed properties. This is confirmed by the loss of significance for curl—BD (Fig 2B) and area–BD (Fig 2C) regressions in the settled treatment. According to previous works (e.g., [18,25,28]), particle characteristics influence the fire behaviour of leaf litter beds by means of controlling the fuel bed structure. Thus, decoupling of the particle characteristics and fuel bed properties should lead to a loss of the relationships between particle characteristics and fire behaviour of the settled leaf litter bed. This has been demonstrated here for curl. In the fresh treatment (Fig 3A, 3D, 3G and 3J) and in previous works [18,34] curl acts as a strong predictor of the fire behaviour of constructed leaf litter beds. However, after subjecting samples to the settled treatment no significant regression between curl and any of the fire behaviour parameters could be found (Fig 3A, 3D, 3G and 3J).

The regression between area and FH also lost its significance in the settled treatment. Even though the regression between area and FD persisted in the settled treatment (Fig 3B), there are strong indications that it was governed by different underlying principles in the fresh and the settled treatment. In the fresh treatment the relationship between area and FD fits previous explanations that an increase in particle size will result in a decrease in BD and an increase in reaction velocity (e.g., [18,25,28,61]). When the reaction velocity increases, the fuel is consumed faster and flames extinguish sooner resulting in a lower FD. Nevertheless, in the settled treatment a short FD can occur in fuel beds composed of large, now densely packed particles in which the flames spread slowly through the upper fuel layer, but do not ignite the densely packed lower fuel layers (Fig 5B). Due to the lower amount of fuel taking part in the flaming combustion, flames extinguish faster and large amounts of unburned material (high UC) are left. In the latter case a low FD is not a result of increased reaction velocity, but rather of slow flaming, a lower amount of combusted fuel and an early flame extinguishment. The generalisation of a significant regression between area and RoS at the whole data set level (Fig 3H) should also be reconsidered since the given regression was driven by one single data point which acted as an influential case (i.e. the exclusion of this point results in an R^2^ of 0.10 and a P-value of 0.293).

The majority of the relationships between particle characteristics and fire behaviour of the leaf litter bed, which were significant in the fresh treatment, lost their significance in the settled treatment. The relationship between area and FD was driven by different underlying principles in the fresh and the settled treatment. Thus, we consider our second hypothesis valid. In contrast to particle characteristics, all relationships which were found between BD and fire behaviour parameters in the fresh treatment were also valid for the whole data set, confirming BD as a stable predictor of FD, FH and RoS [34,48,50,67].

With exception of the curl–UC relationship for the fresh treatment (Fig 3J) and the BD–SD relationship for the settled treatment (Fig 3K), particle/fuel bed characteristics showed significant regressions only with fire behaviour parameters loading highly on the PC1 (Figs 2, 3 and 4). Thus, a large proportion of the overall variation in fire behaviour related to effects on soil (PC2), smouldering combustion (PC3), and the tendency of the material to ignite (PC4) was not explained through changes in the measured particle characteristics or BD.

### Initial bulk density and effects of the settling treatment

In our third hypothesis we postulated that litter beds composed of larger particles with lower initial BD (BD_f_) will be more affected by settling than litter beds composed of smaller particles with higher initial BD. This hypothesis was partially confirmed. Relationships were found between BD_f_ and ΔBD, ΔFD, ΔFH and ΔRoS (Fig 4A, 4C, 4E and 4G) and confirmed that fuel beds with lower BD_f_ are more affected by the settling treatment. Nevertheless, area exhibited significant relationships only with ΔBD and ΔFH (Fig 4B, 4D, 4F and 4G). Thus, BD_f_ had a larger control on the effects of the settled treatment on leaf litter bed fire behaviour than area. This could be attributed to the fact that BD_f_ better describes the fuel bed structure and it is only partially controlled by area (Fig 2C). Curl is also important (Fig 2B) in determining BD_f_ and should be taken into account, especially as it is not always positively related to particle size [34].

The presumed relationship between particle size and initial BD (e.g., BD of samples constructed using dry particles, corresponding to BD_f_) has been previously established (e.g.,[31])and could be confirmed in our study (Fig 2C). The expectation that lower BD_f_ leads to higher effects of settling on the fuel bed is based on the relationship between BD and the packing ratio [34,50,61] (S7 Appendix). If tissue density is disregarded (it covers only a relatively narrow range in our study), a decrease in BD relates to a decrease in the packing ratio [61] and a higher proportion of empty space between the particles. A large proportion of empty space means lower contact surfaces between particles, providing lower structural support to the fuel bed as a whole. If a fuel bed is constructed using dry particles, as in the fresh treatment, the maximum material specific BD will be reached and maintained[28,34,52,66]. However, a loss of rigidity and an increase of particle fresh mass induced by wetting can lead to a further increase of BD, beyond the maximum material specific BD of dry particle litter beds. As BD of a fuel bed increases, the amount of empty space within the fuel bed decreases, the contact surface between particles increases and provides a higher structural support to the fuel bed, resulting in increasing resistance to further compaction. We found a negative logarithmic relationship between BD_f_ and ΔBD (Fig 4A) which confirms that an increase in BD results in a higher resistance to further compaction. Furthermore, the reduction of the ΔBD value with increasing BD_f_ indicates the existence of a threshold BD above which settling alone no longer results in fuel bed compaction. Nevertheless, as the lowest ΔBD in our study was 15.7%, we still lack necessary information to assess what this threshold BD may be. Thus, when comparing our results with previous studies we took BD and particle size range into consideration. Our study was conducted on deciduous hardwoods and covers a range of 5.22–14.95 kg m^-3^ for the BD_f_ values. This range largely coincides with BD-s in previous studies that examined the fire behaviour of North American oaks (5.75–14.42 kg m^-3^[63], 5.28–19.74 kg m^-3^ [18]; BD calculated based on dry mass, fuel area and fuel bed depth provided in the corresponding articles). Furthermore, two of the species which were the ones most influenced by the settled treatment in our study, *Q*. *palustris* and *Q*. *shumardii*, share morphological characteristics (i.e. large, curled and lobed leaves) and exhibit a similar fire behaviour in the fresh treatment (i.e. high flames and short flaming duration) as *Quercus kelloggii* and *Quercus garryana* in [18]. *Q*. *kelloggi* and *Q*.*garryana*, when tested immediately after fuel bed preparation, have been designated to the group with high flammability [18]. These coincidences strongly indicate that fuel bed structure and fire behaviour of some of the North American oaks are very likely to be highly affected by a relatively short exposure to outside weather conditions. This might be especially important for those species that shed their leaves at the end or outside of the fire season. Leaf litter of these species is more likely to burn in the upcoming fire season, after months of exposure to environmental conditions, than shortly after their abscission. Furthermore, as demonstrated by the relationships between BD_f_ and Δ values of FH, RoS and FD (Fig 4C, 4E and 4G), taking our findings into account might be of extreme relevance when assessing fire behaviour and fire behaviour change of leaf litter fuels with low initial BD. In contrast, it can be presumed that fire behaviour of species with leaf particles small enough to create constructed fuels of sufficient density to resist ignition (e.g., *Abies veitchii* Lindley and *Picea abies* (L.) H. Karst.[50]) will be much less affected by exposure to outside weather conditions.

We are only starting to understand the relationships between particle characteristics, fuel bed structure and fire behaviour as affected by exposure to outside weather conditions. Thus, we still lack the knowledge to assess the potential effects of exposure to outside weather conditions on fuel bed structure and fire behaviour change of the species covering most of the plant trait spectrum (e.g., gymnosperms [25], Australian perennial species [34], or species with higher initial BD [27,48,52,68]). Even more so, the size of the effects measured in our study indicates that more attention should be given to the fuel bed dynamics.

### Investigating effects of aging on leaf litter fire behaviour

In their synthesis, [31]identify the effects of decay on fire behaviour of litter beds as one of the issues that deserves more attention in the future. Even though the importance of understanding the relationship between functional traits, decomposition and fire behaviour is recognised [32,69], only few studies have directly investigated the effects of aging on particle characteristics and fire behaviour of litter fuels. In these studies, fuel aging is addressed through repeated sampling of leaf litter [35,42], the time since treatment for masticated fuels [43,44], or the reduction in wood density for twigs [70]. While twigs were tested as individual standardised fragments, leaf litter and masticated fuels were tested as constructed litter beds (i.e. collected as bulk particles, and reconstructed in the laboratory after particle sorting[45]) and tested immediately after construction. Fast and easy collection, the possibility of fuel standardisation, (e.g., testing samples with standardised mass[18,68,71], height or volume [34,45,52], composition [27,48,71–74]) and particle manipulation [25,28], as well as the ability to construct large litter beds (e.g.,[51]) can be considered as advantages of constructed samples. Sampling of aged particles and the subsequent construction of the fuel bed in the laboratory accounts for particle fragmentation [35] and aging induced chemical changes of the fuels [44]. Nevertheless, this procedure does not account for structural changes in the fuel bed addressed in our study. As indicated by our results, individual particles separated from the litter bed complex will regain their curl. If curl is substantial, it is reasonable to presume that destructing the in-stand formed litter bed complex into individual particles and then reconstructing the litter bed in the laboratory may lead to a more uniform and lower BD of the constructed litter bed compared to the in-stand conditions. This reasoning seems contrary to the results reported by [45]. They reported a lower BD, a lower rate of spread and rate of consumption for intact samples (i.e. samples collected in the stand, up to mineral soil, while keeping their composition, structure, compactness and bulk-density intact) compared to the constructed samples. Nevertheless, [45] collected intact samples just after leaf/needle abscission. Thus, it is very likely that newly abscised litter had not yet undergone noticeable compaction, and that the upper layer of the intact sample resembled our fresh treatment. In contrast, their constructed samples were created by using 10 sub-samples collected in each plot and compacting them to match the litter bed depth recorded on-site. The chosen construction method resulted in higher BD of composed compared to intact samples. A s indicated by the authors themselves, the lower rate of spread and rate of consumption can be attributed to artificial compaction of the constructed samples[45].

Furthermore, testing of mass standardised samples in previous studies dealing with leaf litter aging [35,42] does not account for mass loss induced by decomposition. Standardisation can be justified as essential part of experimental work, although it is inevitably reductionistic [75]. Unfortunately, little attention was previously given to effects of sample construction on structural fuel bed properties. Until now, when fire behaviour of leaf litter beds was to be tested in the laboratory, intact samples presented the only alternative to constructed samples [45]. Collection of intact samples requires extra care during sample collection and transport. Intact samples were used for investigating long term effects or fire recurrence [76], substrate [77], vegetation type [78,79] or species [80] on fuel bed structure and fire behaviour. Nevertheless, logistical issues associated with repeated sampling of multiple sites within a short time period make intact samples marginally applicable for investigating short term, settling and decomposition induced changes in fire behaviour of litter beds.

The approach presented here can be seen as a compromise between immediate testing of constructed samples and intact samples. As the initial sample is a constructed sample, the material can be easily collected, and the amounts and composition of the fuel can be standardised and the particle size can be manipulated. Thus, a high variety of fuel mixtures and diverse fuel loads can be tested. In-stand exposure of constructed samples allows for natural fuel bed compaction, weathering and decomposition to take place, affecting not only the fuel bed structure, but also the chemical composition, the fuel load and particle characteristics (S12 Appendix). Exposure of samples to outside conditions results in a fuel bed structure close to that of the intact sample, as demonstrated by the BD gradient observed in the *Q*. *shumardii* samples from the settled treatment (Fig 5). If required, mixtures of different litter fuels can be used to even better approximate in-stand fuel composition [45]. Additionally, the upper side of the exposure construction developed here is left open. Thus, by simply removing the protection net, material can be added to a sample if required[81]. Since each of the four skewer tent pegs, which fix the exposure construction to the ground, also supports the protection net, we did not encounter the potential problem of the protection net sagging and compressing litter material [81].

Furthermore, since materials from various origins can be exposed in a single stand, the requirement for repeated sampling of multiple sites is not necessary. By selecting appropriate exposure conditions and duration the focus can be shifted to the processes of interest. Here we focused on fuel bed settling as induced by 30 days of winter exposure to outside weather conditions. Low temperatures during the exposure period (S2 Appendix) minimised decomposition and leaching. Even though effects of these processes were still measurable (S12 Appendix), settling was a dominant process (minimal ΔBD = 15.7%) with pronounced effects (e.g., Fig 1H) and high explanatory power (e.g., Fig 1H, Fig 4A, 4C, 4E and 4G). Thus we do not address results related to other processes. In a follow-up study (manuscript in preparation), the same approach was successfully applied to test the effects of a single precipitation event (i.e. excluding decomposition) and prolonged spring exposure (i.e. promoting decomposition) on fuel bed structure and resulting fire behaviour.

Conceptually, our approach can be viewed as an expansion of “litter bag” experiments. Whereas common litter bags experiments focus on mass loss and changes in the chemical composition of litter over time in relation to abiotic and biotic factors (e.g.,[37,82]), the method presented here enables further measurements of the changes in the fuel bed structure (e.g., sample height, bulk density, packing ratio) and particle characteristics (e.g., dimensions, density), as well as the resulting fire behaviour. Combining the described methods of fuel exposure and fire behaviour testing with prolonged exposure and successive retrieval of a proportion of the exposed samples (a procedure commonly used in litter bag experiments) would enable direct coupling of decomposition and fire behaviour research. Thus, this could create an opportunity to directly test the validity of previously established conceptual relationships between these two processes essential for carbon cycling [32,33] and bridge the knowledge gap related to the effects of decay on fire behaviour of litter beds [31].

## Conclusions

There is an increasing body of work indicating that particle size and curl, through controlling litter bed aeration, are primary drivers of the leaf litter bed fire behaviour [18,25,27,28,30,34]. Nevertheless, studies indicating this relationship do not account for temporal changes in the litter bed characteristics, most probably as existing methods are not designed to address this issue. Here we present the first results of a method specially developed to quantify the effects of temporal changes in the fuel bed characteristics and fire behaviour of leaf litter beds. We demonstrated that already 30 days of winter exposure to outside weather conditions (settled treatment) was sufficient to decouple the relationship between particle characteristics (curl, area) and fuel bed structure (BD). Consequently, particle characteristics became substantially less reliable predictors of the leaf litter fire behaviour. Furthermore, statistically significant and large effects of the settled treatment on six out of seven measured fire behaviour parameters indicate that fire behaviour of a leaf litter bed can abruptly change even after a relatively short term exposure to winter weather conditions, with larger changes for leaf litter beds with low initial BD. We do not challenge the notion that there is a relationship between leaf traits and fire regime[18,24]. However, our results indicate that, at least for leaf litter with low initial bulk density, this relationship in not necessarily directly driven by the fire behaviour of freshly fallen leaf litter. It can be presumed that deviation of the fire behaviour from that of the freshly fallen leaf litter depends not only on the initial bulk density, but also on the duration of the time period between abscission and fire, as well as on the weather conditions during this time period. Nevertheless, studies with multiple exposure periods are necessary to explore these presumptions. As this is the first experiment of the kind and only a limited number of species was tested, our conclusions can be extrapolated only within the range of the values tested here. Even so, the size of the measured effects indicates that initial, abrupt changes in the fuel bed characteristics deserve more attention in the future.

## Supporting information

S1 Appendix Details of the exposure construction. (PDF)

S2 Appendix Weather conditions during exposure of the settled treatment. (PDF)

S3 Appendix Details of the combustion chamber. (PDF)

S4 Appendix Details of the testing cage and sand frame. (PDF)

S5 Appendix Video frames with maximum flame heights. (PDF)

S6 Appendix Details on measuring maximum flame height. (PDF)

S7 Appendix Relationships between packing ratio, bulk density and particle morphology. (PDF)

S8 Appendix Relationships between packing ratio and fire behaviour characteristics. (PDF)

S9 Appendix An example image of the flattened leaf litter particles. (PDF)

S10 Appendix Sample mean values for area and curl. (PDF)

S11 Appendix Measured values for fuel bed structure characteristics and fire behaviour characteristics referred to in the main article. (PDF)

S12 Appendix Effects of the settling treatment on some additional characteristics. (PDF)

## References

[1] Scott AC, Glasspool IJ. The diversification of Paleozoic fire systems and fluctuations in atmospheric oxygen concentration. Proc Natl Acad Sci. 2006;103(29): 10861–10865. 10.1073/pnas.0604090103 16832054 PMC1544139

[2] Archibald S, Lehmann CER, Belcher CM, Bond WJ, Bradstock RA, Daniau A-L, et al Biological and geophysical feedbacks with fire in the Earth system. Environ Res Lett. 2018;13(3): 033003 10.1088/1748-9326/aa9ead

[3] Liu Y, Goodrick S, Heilman W. Wildland fire emissions, carbon, and climate: Wildfire–climate interactions. For Ecol Manage. 2014;317: 80–96. 10.1016/j.foreco.2013.02.020

[4] Langmann B, Duncan B, Textor C, Trentmann J, van der Werf GR. Vegetation fire emissions and their impact on air pollution and climate. Atmos Environ. 2009;43(1): 107–116. 10.1016/j.atmosenv.2008.09.047

[5] Thelen B, French NHF, Koziol BW, Billmire M, Owen RC, Johnson J, et al Modeling acute respiratory illness during the 2007 San Diego wildland fires using a coupled emissions-transport system and generalized additive modeling. Environ Heal. 2013;12(1):94 10.1186/1476-069X-12-94 24192051 PMC3842653

[6] Wittkuhn RS, Lamont BB, He T. Combustion temperatures and nutrient transfers when grasstrees burn. For Ecol Manage. 2017;399: 179–187. 10.1016/j.foreco.2017.05.037

[7] Pellegrini AFA, Ahlström A, Hobbie SE, Reich PB, Nieradzik LP, Staver AC, et al Fire frequency drives decadal changes in soil carbon and nitrogen and ecosystem productivity. Nature. 2017;553(7687): 194–198. 10.1038/nature24668 29227988

[8] Bond W, Keeley J. Fire as a global ‘herbivore’: the ecology and evolution of flammable ecosystems. Trends Ecol Evol. 2005;20(7): 387–394. 10.1016/j.tree.2005.04.025 16701401

[9] Bowman DMJS, Balch, Artaxo P, Bond WJ, Carlson JM, Cochrane MA, et al Fire in the Earth System. Science 2009;324(5926): 481–484. 10.1126/science.1163886 19390038

[10] Pausas JG, Keeley JE, Schwilk DW. Flammability as an ecological and evolutionary driver. J Ecol. 2017;105(2): 289–297. 10.1111/1365-2745.12691

[11] Bond WJ, Scott AC. Fire and the spread of flowering plants in the Cretaceous. New Phytol 2010;188(4): 1137–1150. 10.1111/j.1469-8137.2010.03418.x 20819174

[12] Pausas JG, Ribeiro E. Fire and plant diversity at the global scale. Glob Ecol Biogeogr. 2017;26(8): 889–897. 10.1111/geb.12596

[13] Bond WJ, Woodward FI, Midgley GF. The global distribution of ecosystems in a world without fire. New Phytol. 2004;165(2): 525–538. 10.1111/j.1469-8137.2004.01252.x 15720663

[14] Balch JK, Brando PM, Nepstad DC, Coe MT, Silvério D, Massad TJ, et al The Susceptibility of Southeastern Amazon Forests to Fire: Insights from a Large-Scale Burn Experiment. Bioscience. 2015;65(9): 893–905. 10.1093/biosci/biv106

[15] Engber EA, Varner JM III, Arguello LA, Sugihara NG. The Effects of Conifer Encroachment and Overstory Structure on Fuels and Fire in an Oak Woodland Landscape. Fire Ecol. 2011;7(2): 32–50. 10.4996/fireecology.0702032

[16] Hessburg PF, Spies TA, Perry DA, Skinner CN, Taylor AH, Brown PM, et al Tamm Review: Management of mixed-severity fire regime forests in Oregon, Washington, and Northern California. For Ecol Manage. 2016;366: 221–250. 10.1016/j.foreco.2016.01.034

[17] Huang Y, Wu S, Kaplan JO. Sensitivity of global wildfire occurrences to various factors in the context of global change. Atmos Environ. 2015;121: 86–92. 10.1016/j.atmosenv.2015.06.002

[18] Engber EA, Varner JM. Patterns of flammability of the California oaks: the role of leaf traits. Can J For Res. 2012;42(11): 1965–1975. 10.1139/x2012-138

[19] Simpson KJ, Ripley BS, Christin P-A, Belcher CM, Lehmann CERR, Thomas GH, et al Determinants of flammability in savanna grass species. J Ecol. 2016 1;104(1): 138–148. 10.1111/1365-2745.12503 26877549 PMC4738432

[20] Wyse S V, Perry GLW, Curran TJ. Shoot-Level Flammability of Species Mixtures is Driven by the Most Flammable Species: Implications for Vegetation-Fire Feedbacks Favouring Invasive Species. Ecosystems. 2018;21(5): 886–900. 10.1007/s10021-017-0195-z

[21] Cóbar-Carranza AJ, García RA, Pauchard A, Peña E. Effect of *Pinus contorta* invasion on forest fuel properties and its potential implications on the fire regime of *Araucaria araucana* and *Nothofagus antarctica* forests. Biol Invasions. 2014;16(11): 2273–2291. 10.1007/s10530-014-0663-8

[22] Fuentes-Ramirez A, Veldman JW, Holzapfel C, Moloney KA. Spreaders, igniters, and burning shrubs: plant flammability explains novel fire dynamics in grass-invaded deserts. Ecol Appl. 2016;26(7): 2311–2322. 10.1002/eap.1371 27755715

[23] Zylstra P, Bradstock RA, Bedward M, Penman TD, Doherty MD, Weber RO, et al Biophysical Mechanistic Modelling Quantifies the Effects of Plant Traits on Fire Severity: Species, Not Surface Fuel Loads, Determine Flame Dimensions in Eucalypt Forests. Bohrer G, editor. PLoS One. 2016;11(8): e0160715 10.1371/journal.pone.0160715 27529789 PMC4986950

[24] Schwilk DW, Caprio AC. Scaling from leaf traits to fire behaviour: community composition predicts fire severity in a temperate forest. J Ecol. 2011 7;99(4): 970–980. 10.1111/j.1365-2745.2011.01828.x

[25] Cornwell WK, Elvira A, van Kempen L, van Logtestijn RSPP, Aptroot A, Cornelissen JHC. Flammability across the gymnosperm phylogeny: the importance of litter particle size. New Phytol. 2015;206(2): 672–681. 10.1111/nph.13317 25675853

[26] Pyne SJ, Andrews PL, Laven RD. Introduction to Wildland Fire. 2nd ed Wiley; 1996.

[27] de Magalhães RMQQ Schwilk DW. Leaf traits and litter flammability: evidence for non-additive mixture effects in a temperate forest. J Ecol. 2012 9;100(5): 1153–1163. 10.1111/j.1365-2745.2012.01987.x

[28] Scarff FR, Westoby M. Leaf litter flammability in some semi-arid Australian woodlands. Funct Ecol. 2006;20(5): 745–752. 10.1111/j.1365-2435.2006.01174.x

[29] Murray BR, Hardstaff LK, Phillips ML. Differences in Leaf Flammability, Leaf Traits and Flammability-Trait Relationships between Native and Exotic Plant Species of Dry Sclerophyll Forest. PLoS One. 2013;8(11): e79205 10.1371/journal.pone.0079205 24260169 PMC3832464

[30] Kauf Z, Fangmeier A, Rosavec R, Španjol Ž. Testing Vegetation Flammability: The Problem of Extremely Low Ignition Frequency and Overall Flammability Score. J Combust. 2014;2014:1–10. 10.1155/2014/970218

[31] Varner JM, Kane JM, Kreye JK, Engber E. The Flammability of Forest and Woodland Litter: a Synthesis. Curr For Reports. 2015;1(2): 91–99. 10.1007/s40725-015-0012-x

[32] Cornelissen JHC, Grootemaat S, Verheijen LM, Cornwell WK, van Bodegom PM, van der Wal R, et al Are litter decomposition and fire linked through plant species traits? New Phytol. 2017;216(3): 653–669. 10.1111/nph.14766 28892160

[33] Dias ATC, Cornelissen JHC, Berg MP. Litter for life: assessing the multifunctional legacy of plant traits. J Ecol. 2017;105(5): 1163–1168. 10.1111/1365-2745.12763

[34] Grootemaat S, Wright IJ, van Bodegom PM, Cornelissen JHC. Scaling up flammability from individual leaves to fuel beds. Oikos. 2017;126(10): 1428–1438. 10.1111/oik.03886

[35] Weir JR, Limb RF. Seasonal Variation in Flammability Characteristics of *Quercus marilandica* and *Quercus stellata* Leaf Litter Burned in the Laboratory. Fire Ecol. 2013;9(3): 80–88. 10.4996/fireecology.0903080

[36] Osono T, Azuma J, Hirose D. Plant species effect on the decomposition and chemical changes of leaf litter in grassland and pine and oak forest soils. Plant Soil. 2014;376(1–2): 411–421. 10.1007/s11104-013-1993-5

[37] Berg B. Decomposition patterns for foliar litter–A theory for influencing factors. Soil Biol Biochem. 2014;78: 222–232. 10.1016/j.soilbio.2014.08.005

[38] Kheirallah AM. Fragmentation of leaf litter by a natural population of the millipede *Julus scandinavius* (Latzel 1884). Biol Fertil Soils. 1990;10(3): 202–206. 10.1007/BF00336137

[39] Lecerf A. Methods for estimating the effect of litterbag mesh size on decomposition. Ecol Modell. 2017;362: 65–68. 10.1016/j.ecolmodel.2017.08.011

[40] Van Stan JT, Coenders-Gerrits M, Dibble M, Bogeholz P, Norman Z. Effects of phenology and meteorological disturbance on litter rainfall interception for a *Pinus elliottii* stand in the Southeastern United States. Hydrol Process. 2017;31(21): 3719–3728. 10.1002/hyp.11292

[41] Hyde JC, Smith AMS, Ottmar RD. Properties affecting the consumption of sound and rotten coarse woody debris in northern Idaho: a preliminary investigation using laboratory fires. Int J Wildl Fire. 2012;21(5): 596–608. 10.1071/WF11016

[42] Kauf Z, Fangmeier A, Rosavec R, Španjol Ž. Seasonal and Local Differences in Leaf Litter Flammability of Six Mediterranean Tree Species. Environ Manage. 2015;55(3): 687–701. 10.1007/s00267-014-0427-3 25537154

[43] Kreye JK, Varner JM, Kane JM, Knapp EE, Reed WP. The impact of aging on laboratory fire behaviour in masticated shrub fuelbeds of California and Oregon, USA. Int J Wildl Fire. 2016;25(9):1002–1008. 10.1071/WF15214

[44] Sikkink PG, Jain TB, Reardon J, Heinsch FA, Keane RE, Butler B, et al Effect of particle aging on chemical characteristics, smoldering, and fire behavior in mixed-conifer masticated fuel. For Ecol Manage. 2017;405: 150–165. 10.1016/j.foreco.2017.09.008

[45] Ganteaume A, Jappiot M, Curt T, Lampin C, Borgniet L. Flammability of litter sampled according to two different methods: comparison of results in laboratory experiments. Int J Wildl Fire. 2014; 23(8): 1061–1075. 10.1071/WF13045

[46] Anderson HE. Forest fuel ignitibility. Fire Technol. 1970; 6(4): 312–319. 10.1007/BF02588932

[47] Martin RE, Gordon DA, Gutierrez ME, Lee DS, Molina DM, Schroeder RA, et al. Assessing the flammability of domestic and wildland vegetation. In: Proceedings of the 12th Conference on Fire and Forest Meteorology. Society of American Foresters; 1994. p. 130–7.

[48] van Altena C, van Logtestijin RSP, Cornwell WK, Cornelissen JHC. Species composition and fire: non-additive mixture effects on ground fuel flammability. Front Plant Sci. 2012;3: Article number 63. 10.3389/fpls.2012.00063 22639656 PMC3355679

[49] Prior L, Murphy B, Bowman D. Conceptualizing Ecological Flammability: An Experimental Test of Three Frameworks Using Various Types and Loads of Surface Fuels. Fire. 2018;1(1):14 10.3390/fire1010014

[50] Zhao W, Cornwell WK, van Pomeren M, van Logtestijn RSP, Cornelissen JHC. Species mixture effects on flammability across plant phylogeny: the importance of litter particle size and the special role for non- Pinus Pinaceae. Ecol Evol. 2016;6(22): 8223–8234. 10.1002/ece3.2451 27878090 PMC5108272

[51] Madrigal J, Guijarro M, Hernando C, Díez C, Marino E. Estimation of Peak Heat Release Rate of a Forest Fuel Bed in Outdoor Laboratory Conditions. J Fire Sci. 2011;29(1): 53–70. 10.1177/0734904110373890

[52] Plucinski MP, Anderson WR. Laboratory determination of factors influencing successful point ignition in the litter layer of shrubland vegetation. Int J Wildl Fire. 2008;17(5): 628–637. 10.1071/WF07046

[53] Rasband WS. Image J. U.S. National Institutes of Health, Bethesda, Maryland, USA; Available from: https://imagej.nih.gov/ij/

[54] Gagnon PR, Passmore HA, Slocum M, Myers JA, Harms KE, Platt WJ, et al Fuels and fires influence vegetation via above- and belowground pathways in a high-diversity plant community. J Ecol. 2015;103(4):1009–19. 10.1111/1365-2745.12421

[55] Santín C, Doerr SH, Merino A, Bryant R, Loader NJ. Forest floor chemical transformations in a boreal forest fire and their correlations with temperature and heating duration. Geoderma. 2016;264: 71–80. 10.1016/j.geoderma.2015.09.021

[56] Chambers DP, Attiwill PM. The ash-bed effect in *Eucalyptus regnans* forest: chemical, physical and microbiological changes in soil after heating or partial sterilisation. Aust J Bot. 1994;42(6):739–49. 10.1071/BT9940739

[57] Emery SM, Uwimbabazi J, Flory SL. Fire intensity effects on seed germination of native and invasive Eastern deciduous forest understory plants. For Ecol Manage. 2011;261(8):1401–8. 10.1016/j.foreco.2011.01.024

[58] Pérez-Harguindeguy N, Díaz S, Garnier E, Lavorel S, Poorter H, Jaureguiberry P, et al New handbook for standardised measurement of plant functional traits worldwide. Aust J Bot 2013;61(3): 167–234. 10.1071/BT12225

[59] Cohen J. A power primer. Psychol Bull. 1992;112(1): 155–159. 10.1037/0033-2909.112.1.155 19565683

[60] Hair JF, Black WC, Babin BJ, Anderson RE. Multivariate Data Analysis: A Global Perspective. Pearson; 2010.

[61] Rothermel RC. A mathematical model for predicting fire spread in wildland fuels. US Dep Agric For Serv Gen Tech Rep. 1972; (INT-115). Available from: https://www.fs.fed.us/rm/pubs_int/int_rp115.pdf

[62] R Core Team. R: A Language and Environment for Statistical Computing. Vienna, Austria: R Foundation for Statistical Computing; 2017 Available from: https://www.r-project.org

[63] Kane JM, Varner JM, Hiers JK. The burning characteristics of southeastern oaks: Discriminating fire facilitators from fire impeders. For Ecol Manage. 2008;256(12): 2039–2045. 10.1016/j.foreco.2008.07.039

[64] Blauw LG, Wensink N, Bakker L, van Logtestijn RSP, Aerts R, Soudzilovskaia NA, et al Fuel moisture content enhances nonadditive effects of plant mixtures on flammability and fire behavior. Ecol Evol. 2015;5(17): 3830–3841. 10.1002/ece3.1628 26380709 PMC4567884

[65] Kreye JK, Varner JM, Hamby GW, Kane JM. Mesophytic litter dampens flammability in fire-excluded pyrophytic oak-hickory woodlands. Ecosphere. 2018;9(1): e02078 10.1002/ecs2.2078

[66] Burrows ND. Fire behaviour in jarrah forest fuels: 1. Laboratory experiments. CALMScience. 1999;3(1): 31–56.

[67] Clarke PJ, Prior LD, French BJ, Vincent B, Knox KJE, Bowman DMJS. Using a rainforest-flame forest mosaic to test the hypothesis that leaf and litter fuel flammability is under natural selection. Oecologia. 2014;176(4): 1123–1133. 10.1007/s00442-014-3071-y 25234374

[68] Prior LD, Murphy BP, Williamson GJ, Cochrane MA, Jolly WM, Bowman DMJS. Does inherent flammability of grass and litter fuels contribute to continental patterns of landscape fire activity? J Biogeogr. 2017;44(6): 1225–1238. 10.1111/jbi.12889

[69] Grootemaat S, Wright IJ, van Bodegom PM, Cornelissen JHC, Cornwell WK. Burn or rot: leaf traits explain why flammability and decomposability are decoupled across species. Funct Ecol. 2015;29(11): 1486–1497. 10.1111/1365-2435.12449

[70] Zhao W, Blauw L, van Logtestijn R, Cornwell W, Cornelissen J. Interactions between Fine Wood Decomposition and Flammability. Forests. 2014;5(4):827–846. 10.3390/f5040827

[71] Varner JM, Kuljian HG, Kreye JK. Fires without tanoak: the effects of a non-native disease on future community flammability. Biol Invasions. 2017;19(8):2307–2317. 10.1007/s10530-017-1443-z

[72] Dickinson MB, Johnson EA, Artiaga R. Fire spread probabilities for experimental beds composed of mixedwood boreal forest fuels. Can J For Res. 2013;43(4):321–330. 10.1139/cjfr-2012-0291

[73] Ormeño E, Céspedes B, Sánchez IA, Velasco-García A, Moreno JM, Fernandez C, et al The relationship between terpenes and flammability of leaf litter. For Ecol Manage. 2009;257(2):471–482. 10.1016/j.foreco.2008.09.019

[74] Zimmer HC, Auld TD, Hughes L, Offord CA, Baker PJ. Fuel flammability and fire responses of juvenile canopy species in a temperate rainforest ecosystem. Int J Wildl Fire. 2015;24(3):349–360. 10.1071/WF14054

[75] Pausas JG, Moreira B. Flammability as a biological concept. New Phytol. 2012;194(3): 610–613. 10.1111/j.1469-8137.2012.04132.x 22489901

[76] Ganteaume A, Marielle J, Corinne L-M, Thomas C, Laurent B. Effects of vegetation type and fire regime on flammability of undisturbed litter in Southeastern France. For Ecol Manage. 2011;261(12): 2223–2231. 10.1016/j.foreco.2010.09.046

[77] Curt T, Schaffhauser A, Borgniet L, Estève R, Ganteaume A, Jappiot M, et al. Litter flammability of French Mediterranean vegetation types: a cross-substratum analysis. In: Viegas DX, editor. VI International Conference on Forest Fire Research 2010. Available from: https://hal.archives-ouvertes.fr/hal-00583411/document.

[78] Curt T, Schaffhauser A, Borgniet L, Dumas C, Estève R, Ganteaume A, et al Litter flammability in oak woodlands and shrublands of southeastern France. For Ecol Manage. 2011;261(12): 2214–2222. 10.1016/j.foreco.2010.12.002

[79] Ganteaume A, Lampin-Maillet C, Guijarro M, Hernando C, Jappiot M, Fonturbel T, et al Spot fires: fuel bed flammability and capability of firebrands to ignite fuel beds. Int J Wildl Fire. 2009;18(8): 951–969. 10.1071/WF07111

[80] Ganteaume A, Jappiot M, Lampin C. Assessing the flammability of surface fuels beneath ornamental vegetation in wildland-urban interfaces in Provence (south-eastern France). Int J Wildl Fire. 2013;22(3): 333–342. 10.1071/WF12006

[81] Dickinson MB, Hutchinson TF, Dietenberger M, Matt F, Peters MP. Litter Species Composition and Topographic Effects on Fuels and Modeled Fire Behavior in an Oak-Hickory Forest in the Eastern USA. Yang J, editor. PLoS One. 2016;11(8): 10.1371/journal.pone.0159997 27536964 PMC4990339

[82] Fioretto A, Papa S, Pellegrino A, Fuggi A. Decomposition dynamics of Myrtus communis and Quercus ilex leaf litter: Mass loss, microbial activity and quality change. Appl Soil Ecol. 2007;36(1): 32–40. 10.1016/j.apsoil.2006.11.006

