# Supplementary material for: Do relationships between leaf traits and fire behaviour of leaf litter beds persist in time?
Source: PLoS One. 2018 Dec 26;13(12):e0209780. doi: 10.1371/journal.pone.0209780 (PMC6306239; doi:10.1371/journal.pone.0209780)
Supplement: S1 Appendix — (PDF) [file pone.0209780.s001.pdf]

S1 Appendix. Details of the exposure construction

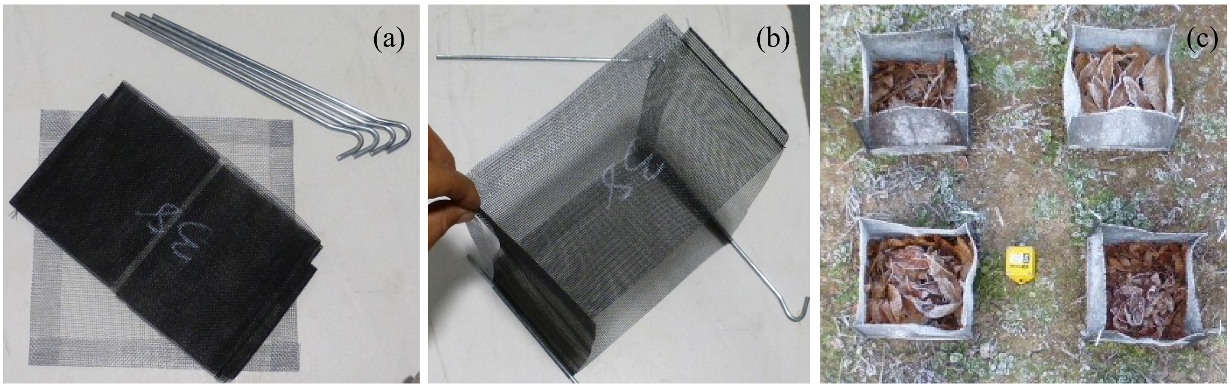

(a) Individual components of the sample exposure construction in the folded state: aluminium bottom, fabric enclosure and 4 tent pegs. When individual components are folded, the volume required for storage of the exposure construction is minimized and the construction can be easily transported without damaging or bending individual elements. All bottoms and fabric enclosures were numbered to ensure sample traceability. The bottom is made of an aluminium wire mesh (wire thickness: 0.21 mm, mesh grid: 2.05 mm x 1.40 mm), the fabric enclosure is made of fiberglass reinforced PVC mesh (fibre diameter: 0.28mm, grid size: 1.2 mm x 1.4 mm). The fabric enclosure has 4 shafts for tent pegs at 20 cm distance. Each shaft is shortened for 2.5 cm at the lower side, thus the fabric enclosure fits perfectly into the aluminium bottom. **(b)** An unfolded exposure construction. The side walls of the aluminium bottom are lifted and the fabric enclosure is put in place. In the photography two of the tent pegs are in the shafts of the fabric enclosure. During installation tent pegs are hammered in the soil so that only the hook stays above the fabric enclosure. **(c)** Four exposed samples. The fabric enclosures seem white due to frost formation. Yellow: TGP-4017 data logger (Tinytag, Gemini Dataloggers Ltd., Chichester, UK), recording temperature at the soil surface. The sample exposure constructions were placed 20 cm apart.
