# Supplementary material for: Do relationships between leaf traits and fire behaviour of leaf litter beds persist in time?
Source: PLoS One. 2018 Dec 26;13(12):e0209780. doi: 10.1371/journal.pone.0209780 (PMC6306239; doi:10.1371/journal.pone.0209780)
Supplement: S2 Appendix — (PDF) [file pone.0209780.s002.pdf]

S2 Appendix. Weather conditions during exposure of the settled treatment.

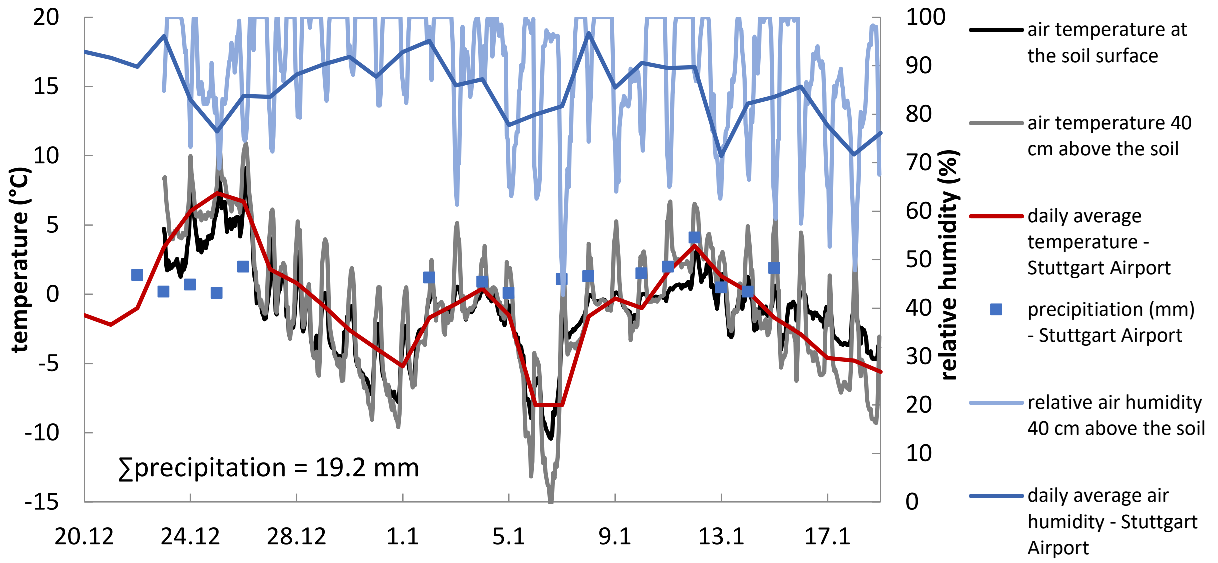

Air temperature at the soil surface was measured with a Tinytag TGP-4017 (Gemini Dataloggers Ltd., Chichester, UK) in the center of the experimental surface. Air temperature and relative humidity 40 cm above the surface were measured with a Tinytag TGU-4500 (Gemini Dataloggers Ltd., Chichester, UK) positioned at the edge of the experimental area. Information on precipitation, daily average temperature and air humidity for Stuttgart Airport are provided by the German Weather Service (Deutscher Wetterdienst, www.dwd.de). Information from the German Weather Service are included to provide the data for the first three days of exposure, when data loggers were not yet installed. Stuttgart Airport is a measuring station close to the experimental area.
