# Supplementary material for: Do relationships between leaf traits and fire behaviour of leaf litter beds persist in time?
Source: PLoS One. 2018 Dec 26;13(12):e0209780. doi: 10.1371/journal.pone.0209780 (PMC6306239; doi:10.1371/journal.pone.0209780)
Supplement: S3 Appendix — (PDF) [file pone.0209780.s003.pdf]

S3 Appendix. Details of the combustion chamber.

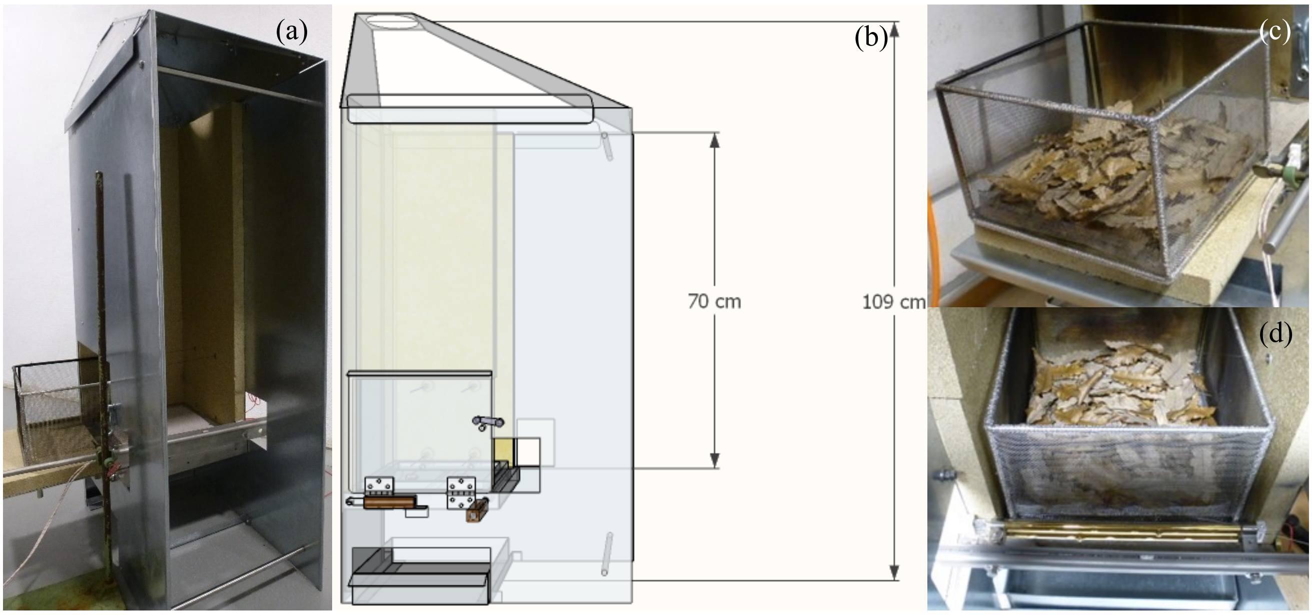

(**a**) Photo of the combustion chamber, with opened side doors on which an empty testing cage is positioned. The frame within the chamber is filled with washed and size calibrated (0.3 - 0.8 mm) dry quartz sand. The IR lamp (1000W, 1.4-1.7 µm, 90 kW/m², with golden reflector, SRSystems GmbH, Bruchköbel, Germany) is in place. **(b)** In scale drawing of the combustion chamber (height x length x width = 109 cm x 52.6 cm x 28.8 cm). The inner walls of the chamber, next to the testing area, are insulated with 2.5 cm thick vermiculite insulation board (V-1100 (700), Skamol, Nykøbing Mors, Denmark). The insulation material is yellow in the drawing. The closable side doors, the platform with flappable bottom and the 2 cm high frame positioned inside the testing chamber, and the opening for the lamp can be seen as well. **(c)** Positioning of a sample (fresh treatment, Lebanon oak (*Quercus libani* Olivier)) into the combustion chamber, on top of the sand surface. The sample is in the testing cage. **(d)** *Q. libani* fresh treatment sample in the testing position. The IR lamp can be seen next to the sample. One of the six capillaries through which thermocouples are placed into position is visible on the right chamber wall. The thermocouple (N-Type, ungrounded, 0.5 mm diameter, TJC1-NNIN-IM050U-150, Omega Engineering) is in place but cannot be seen due to its small diameter.
