# Supplementary material for: Do relationships between leaf traits and fire behaviour of leaf litter beds persist in time?
Source: PLoS One. 2018 Dec 26;13(12):e0209780. doi: 10.1371/journal.pone.0209780 (PMC6306239; doi:10.1371/journal.pone.0209780)
Supplement: S4 Appendix — (PDF) [file pone.0209780.s004.pdf]

S4 Appendix. Details of the testing cage and sand frame.

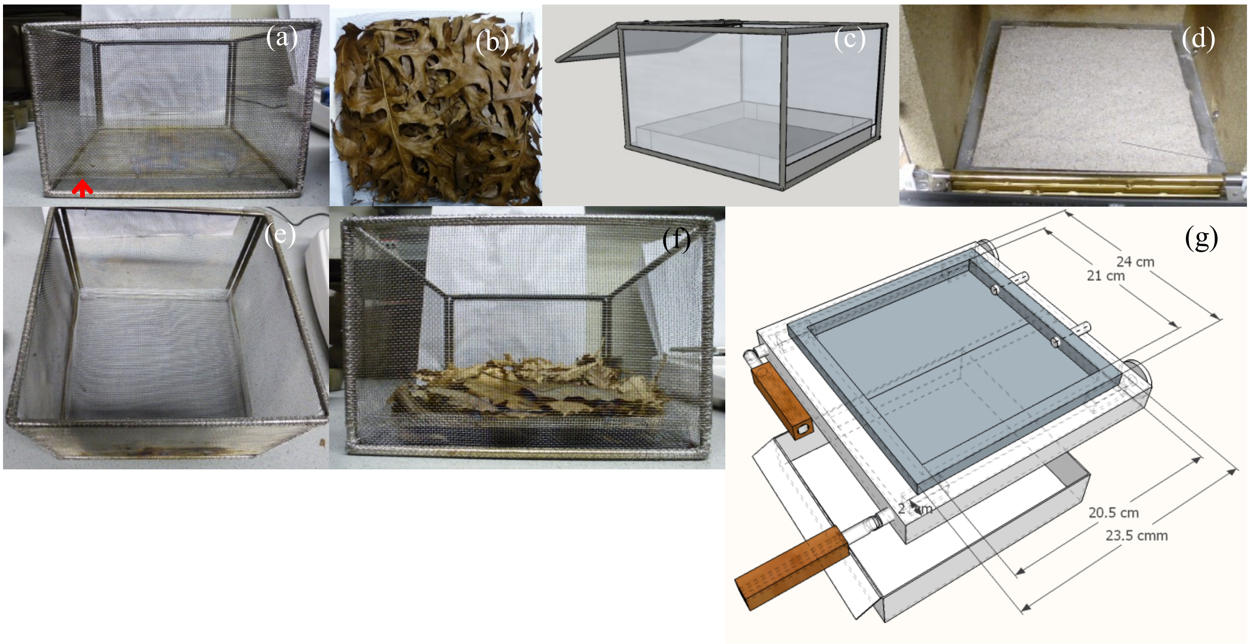

(**a**) Photo of the testing cage. The testing cage has a height of 14.5 cm and an area of 21.0 x 21.5 cm^2. The testing cage is covered with a fine stainless steel mash (V2A steel, wire thickness: 0.21 mm, mesh grid: 1.50 mm x 1.50 mm) and can be flipped open at the back side allowing settled treatment samples to be gently slid inside. The lower 2 cm of its front side are left uncovered to minimize overlapping of the testing cage mesh and the aluminium bottom of the exposure construction (indicated with the red arrow). **(b)** Photo of the settled samples (unique code 43QS-7) before being inserted into the testing cage. The fabric enclosure of the exposure construction is already removed. **(c)** In scale drawing of the testing cage with aluminium bottom slid inside. The back side is fixed to the rest of the testing cage with two strings, thus it can be flipped open and a settled treatment sample can be slid inside. The area of the testing cage is larger than the area of the aluminium bottom of the exposure construction (21.0 x 21.5 cm2 vs. 20.0 x 20.0 cm2), thus settled samples can slide inside without disturbing their structure. **(d)** Photo of the prepared sand surface on which samples are positioned when tested (also visible in the S1.2. a and c). **(e)** Photo of the testing cage ready for construction of the fresh treatment sample. To insure the same testing condition the same type of the aluminium bottom as the ones used for the exposure construction (S1.1.) is placed in the testing cage before a fresh treatment sample is constructed. **(f)** Photo of the front view of a prepared fresh treatment sample of Lebanon oak (*Quercus libani* Olivier). The area in which the aluminium bottom and the stainless steel mesh of the testing cage overlap is visible. **(g)** In scale drawing of the platform with the flappable bottom and the 2 cm high frame which are inseparable part of the testing chamber. The sand collection drawer is also visible. In the chamber insulation material surrounds the sand frame (grey in the drawing) from three sides, the front side is left uninsulated. The bottom of the sand frame can be flipped open enabling quick exchange of the sand between tests.
