# Supplementary material for: Do relationships between leaf traits and fire behaviour of leaf litter beds persist in time?
Source: PLoS One. 2018 Dec 26;13(12):e0209780. doi: 10.1371/journal.pone.0209780 (PMC6306239; doi:10.1371/journal.pone.0209780)
Supplement: S5 Appendix — (PDF) [file pone.0209780.s005.pdf]

S5 Appendix. Video frames with maximum flame heights.

Species: European ash
(*Fraxinus excelsior* L.)

Treatment:	Replicate:	1	2	3	4	5
fresh		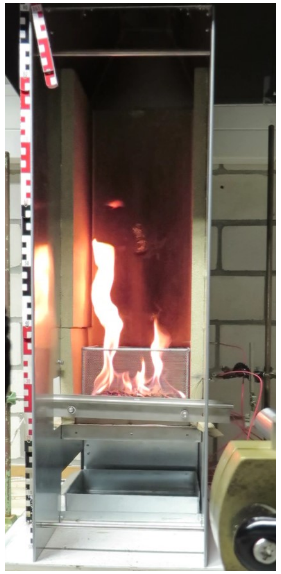	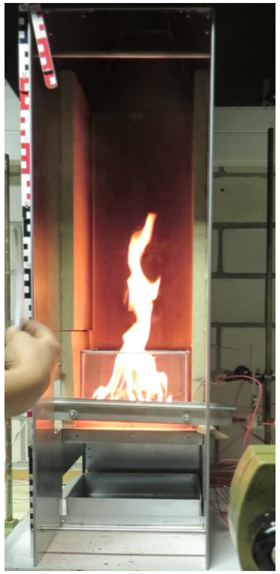	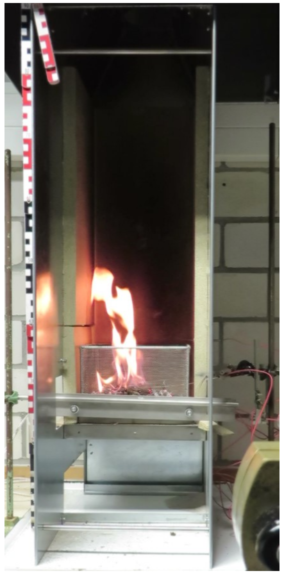	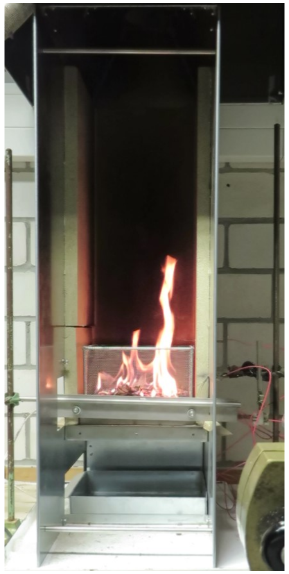	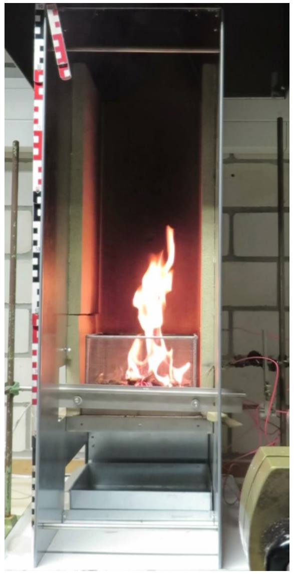
settled		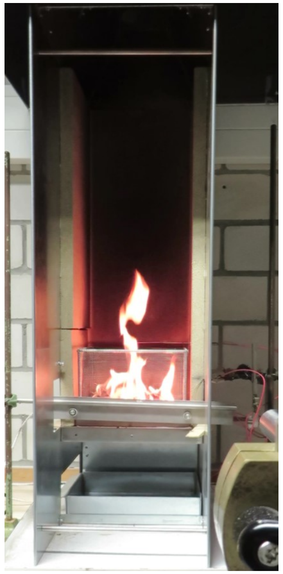	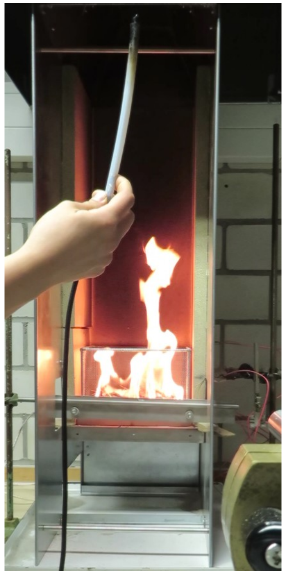	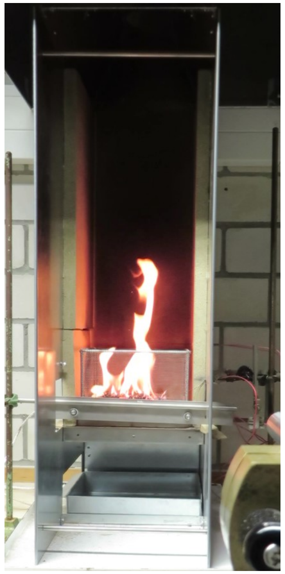	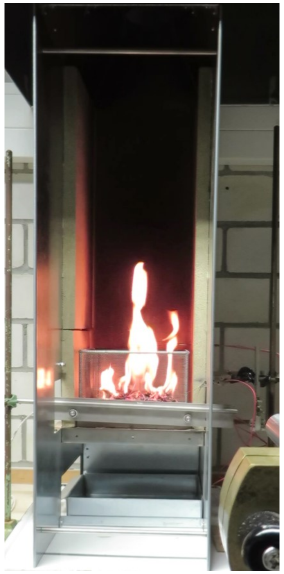	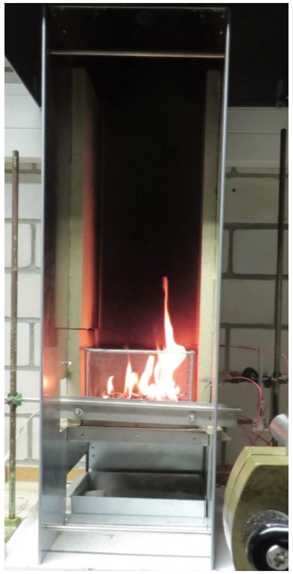

Species: Manchurian ash
(*F. mandshurica* Rupr.)

Treatment:	Replicate:	1	2	3	4	5
fresh		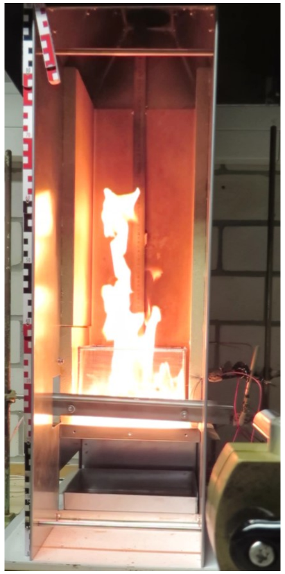	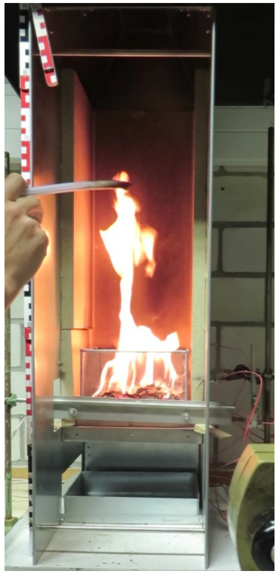	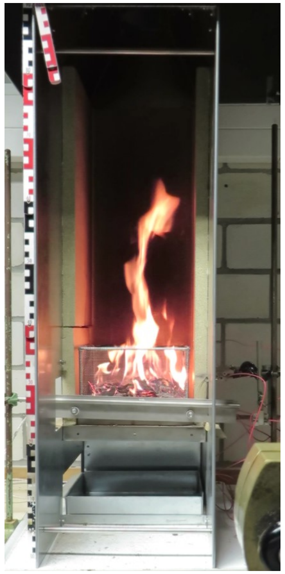	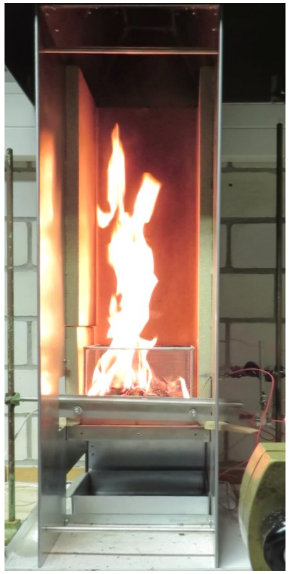	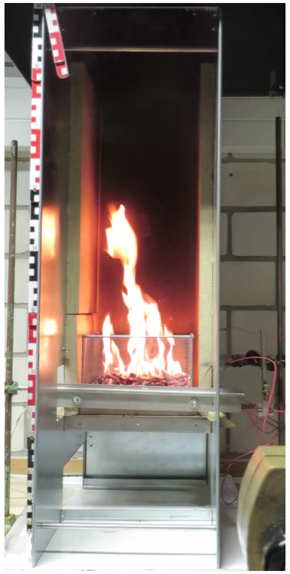
settled		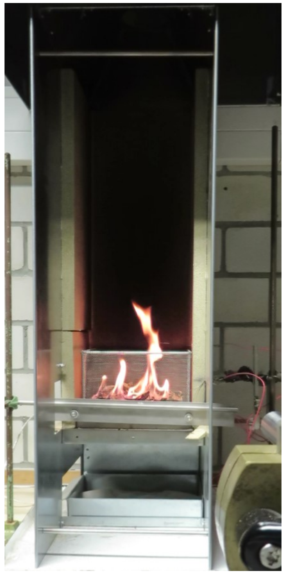	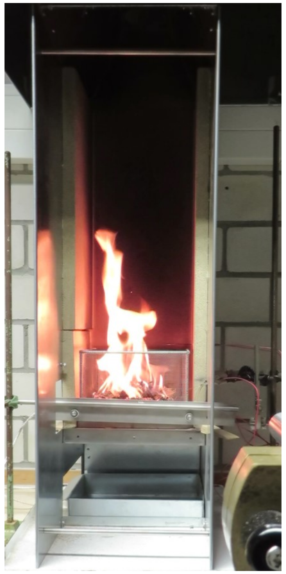	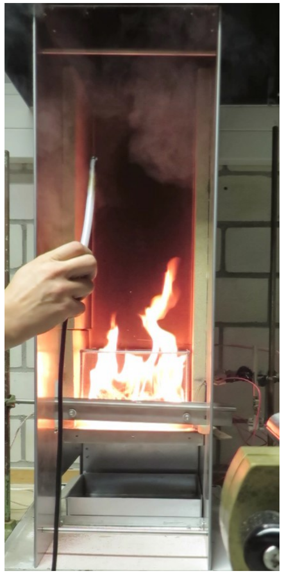	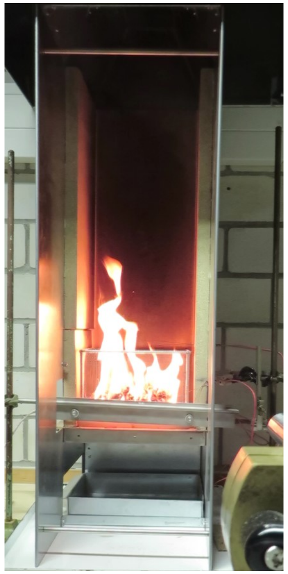	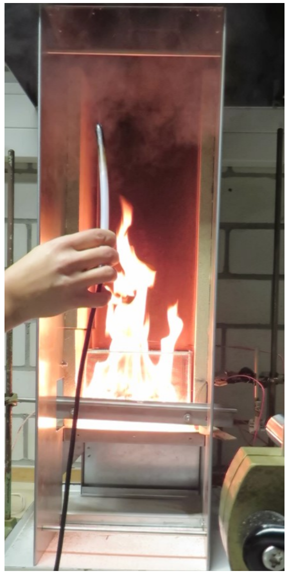

Species: Shingle oak
(*Quercus imbricaria* Michx.)

Treatment:	Replicate:	1	2	3	4	5
fresh		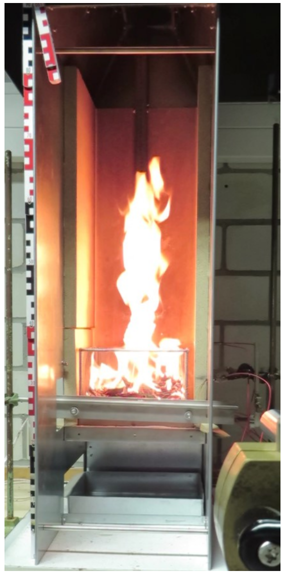	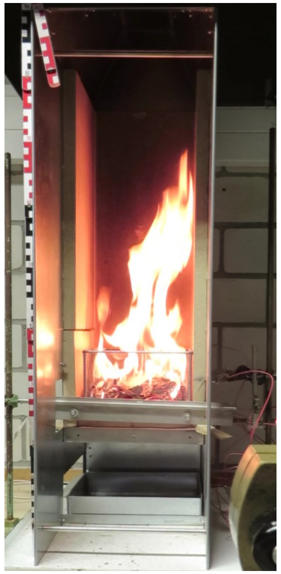	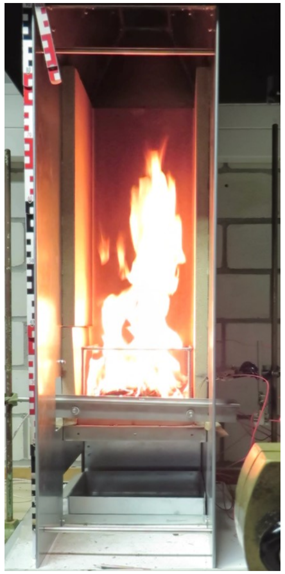	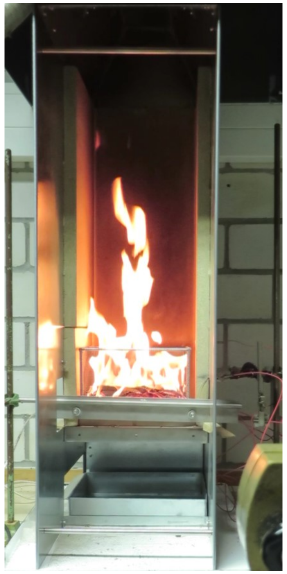	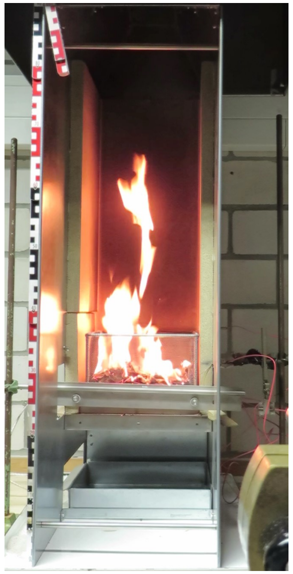
settled		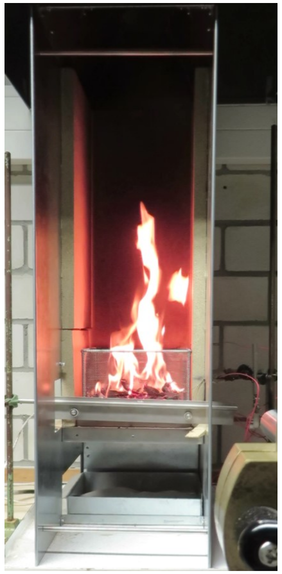	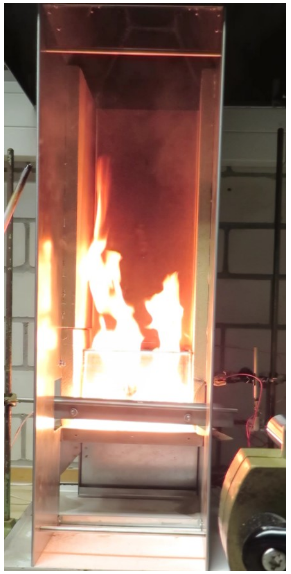	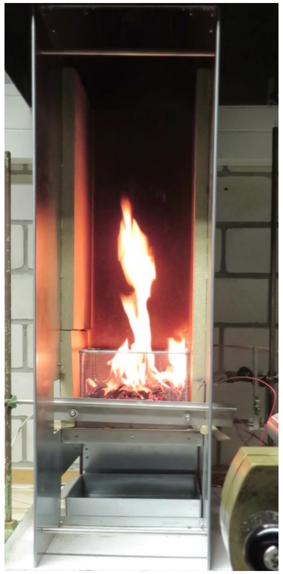	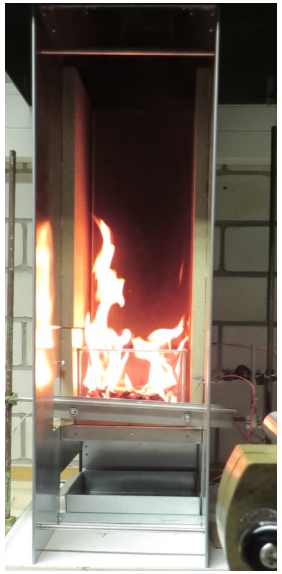	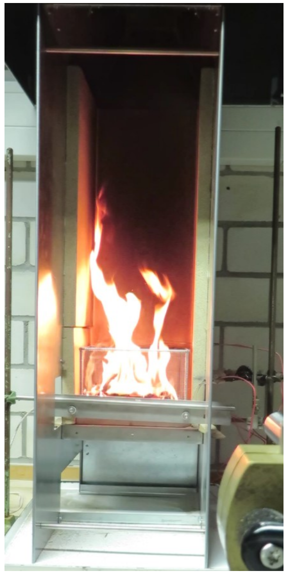

Species: Lebanon oak
(*Q. libani* Olivier)

Treatment:	Replicate:	1	2	3	4	5
fresh		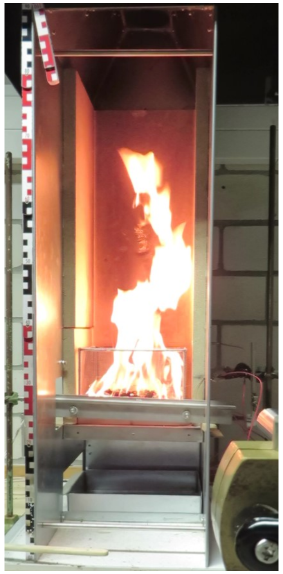	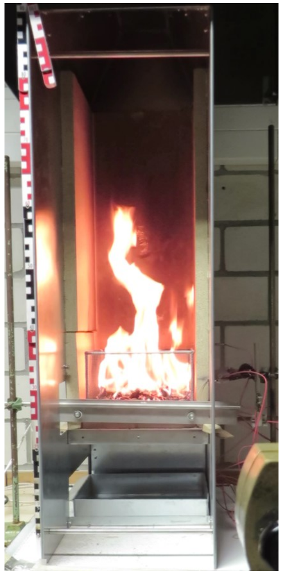	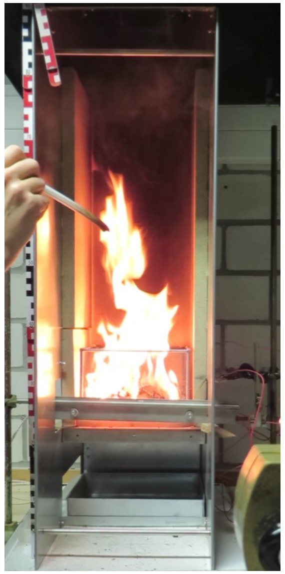	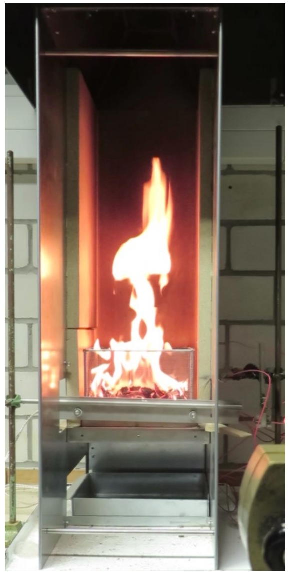	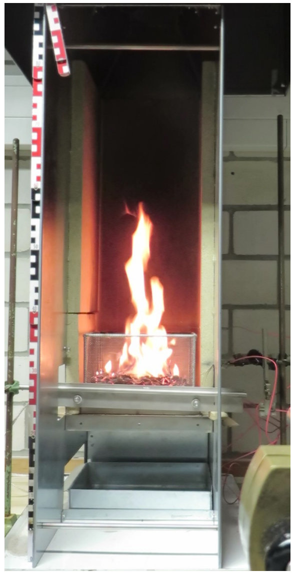
settled		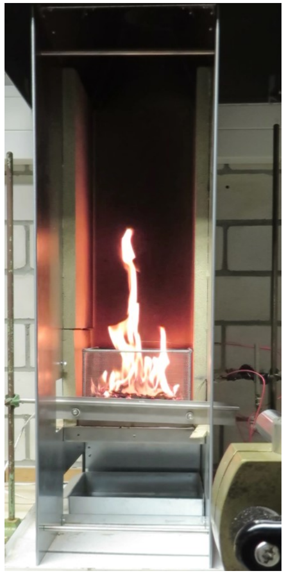	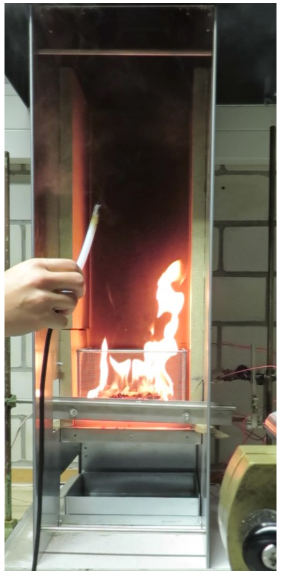	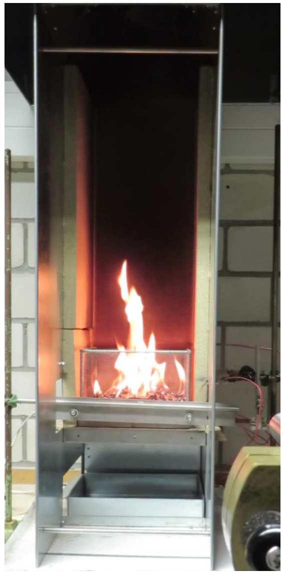	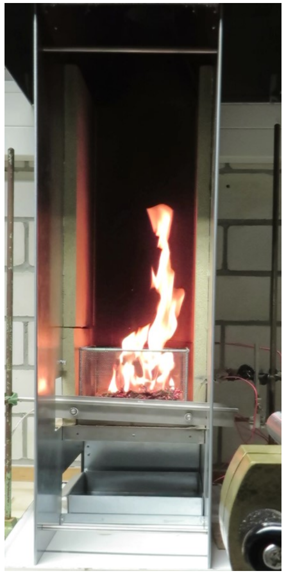	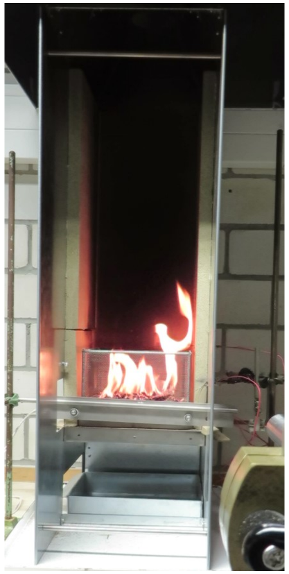

Species: Pin oak
(*Q. palustris* Münchh.)

Treatment:	Replicate:	1	2	3	4	5
fresh		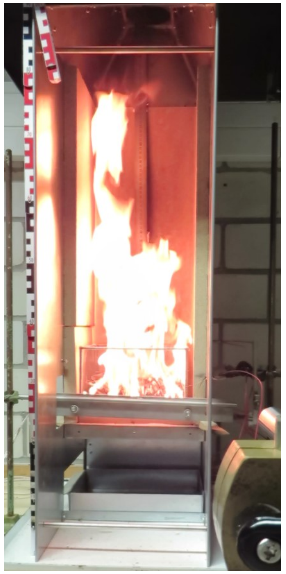	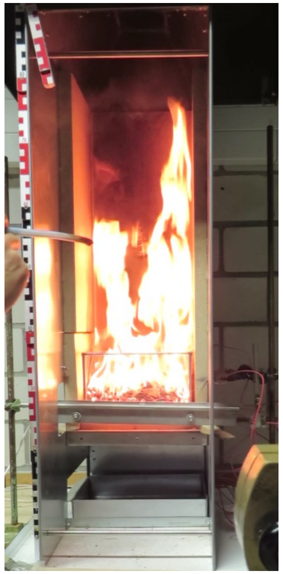	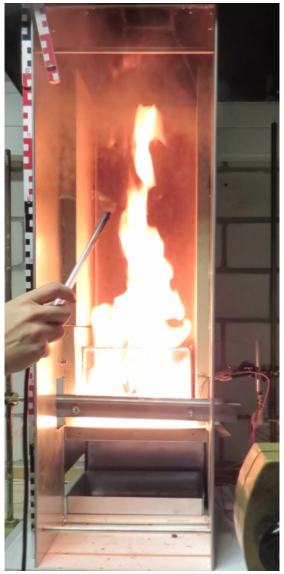	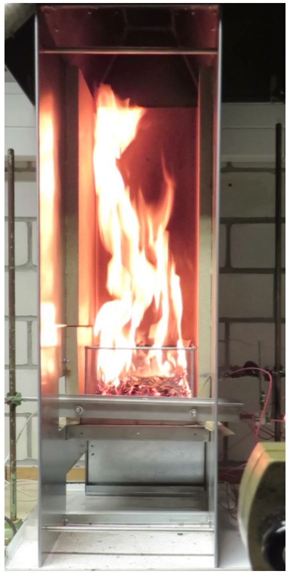	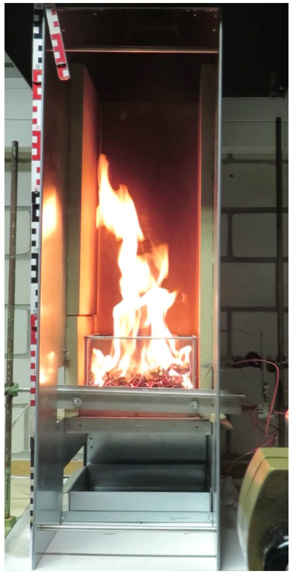
settled		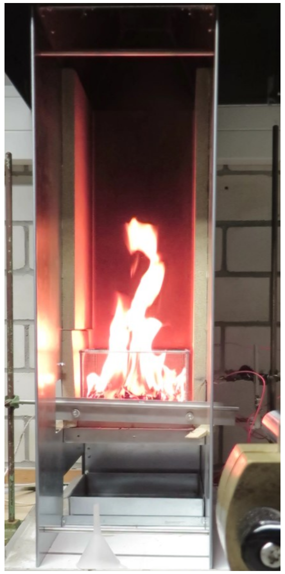	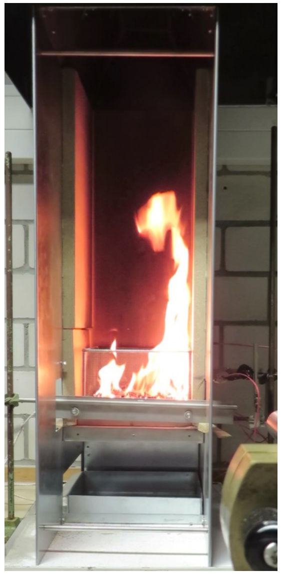	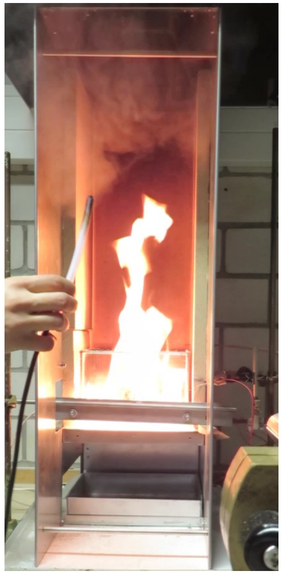	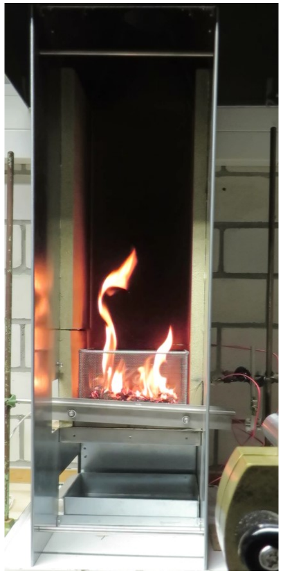	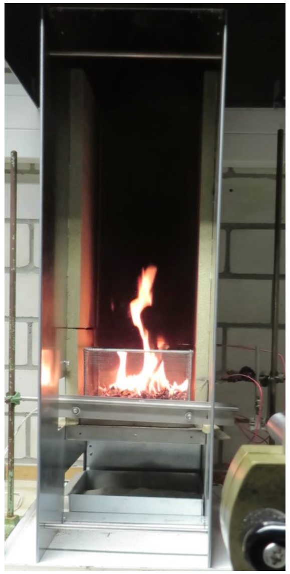

Species: Spotted oak
(*Q. shumardii* Buckl.)

Treatment:	Replicate:	1	2	3	4	5
fresh		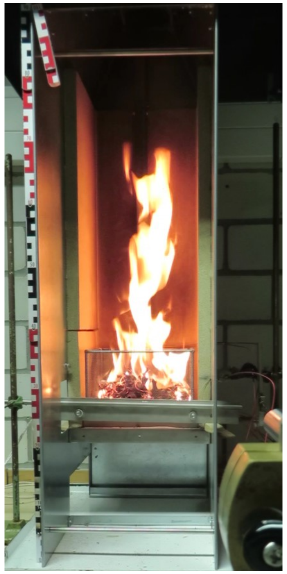	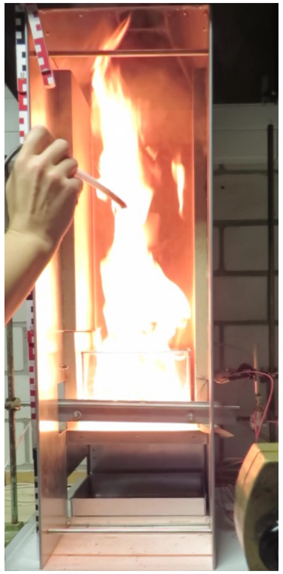	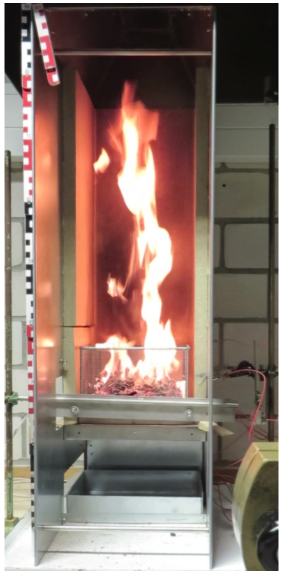	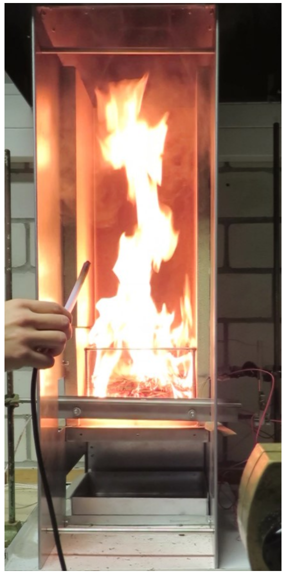	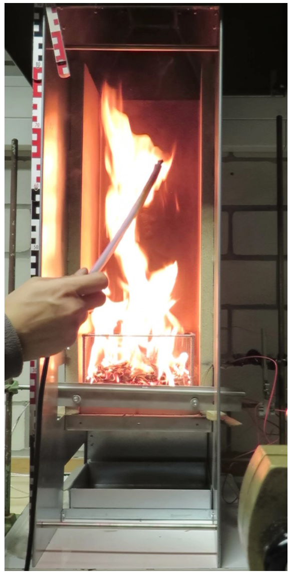
settled		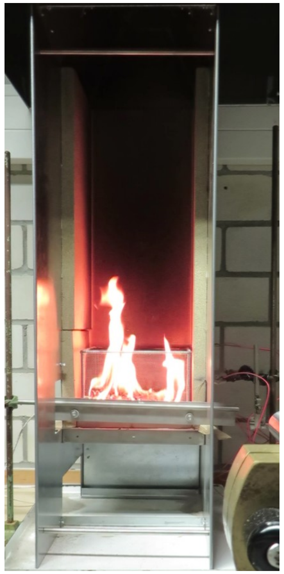	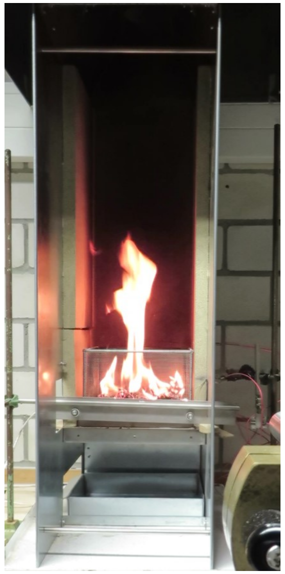	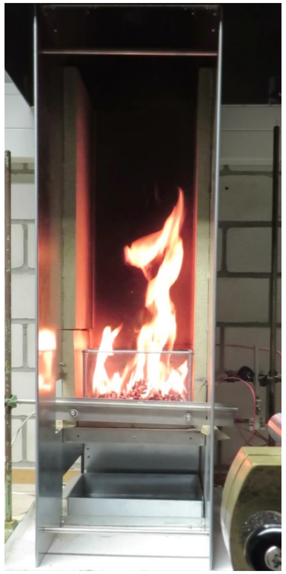	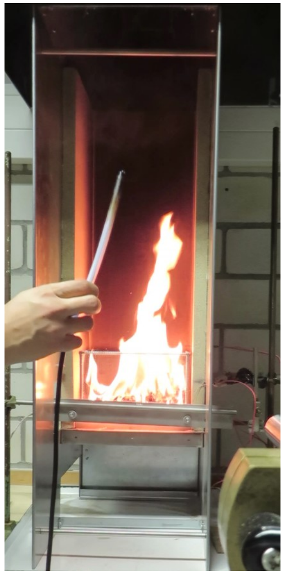	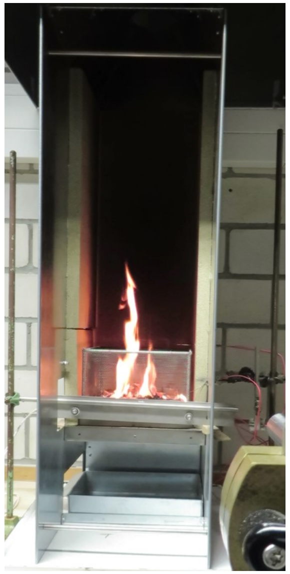

Species: Service tree
(*Sorbus domestica* L.)

Treatment:	Replicate: 1	2	3	4	5
fresh	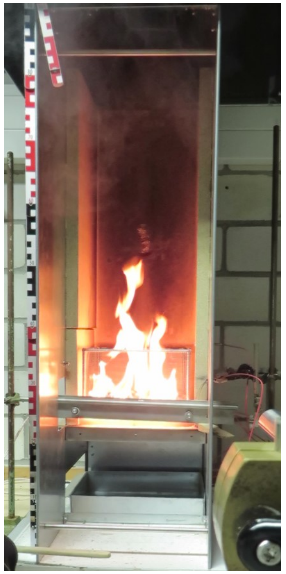	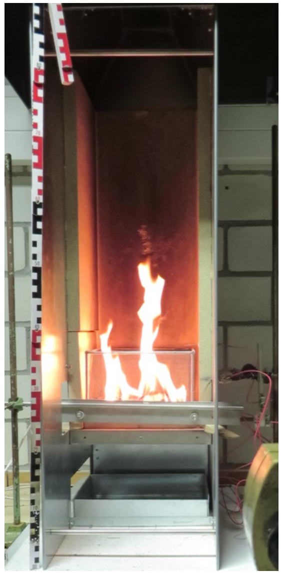	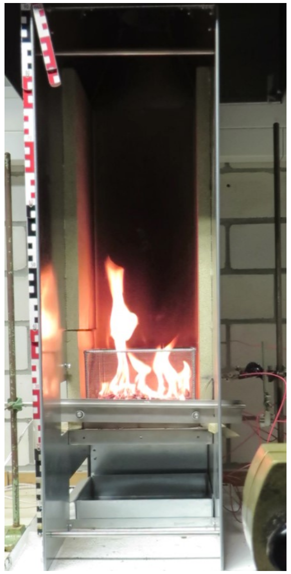	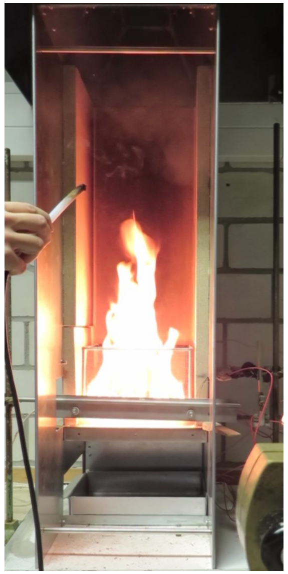	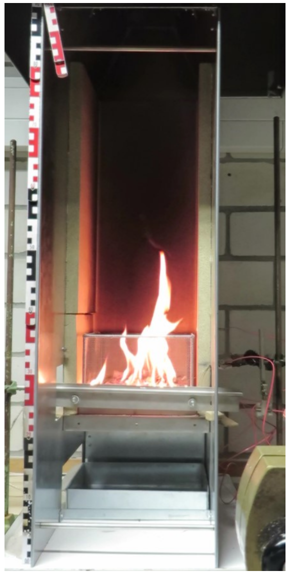
settled	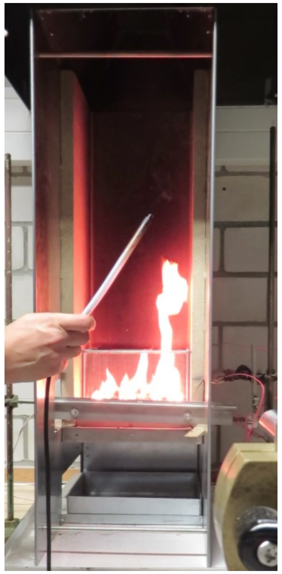	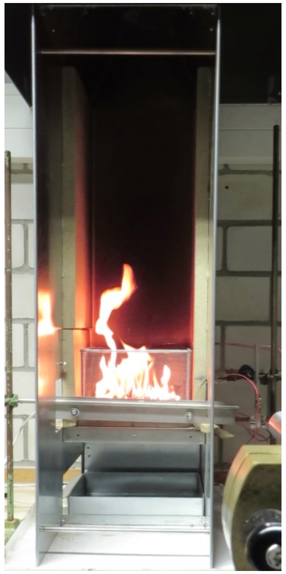	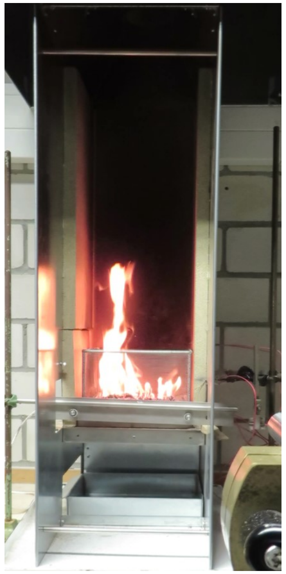	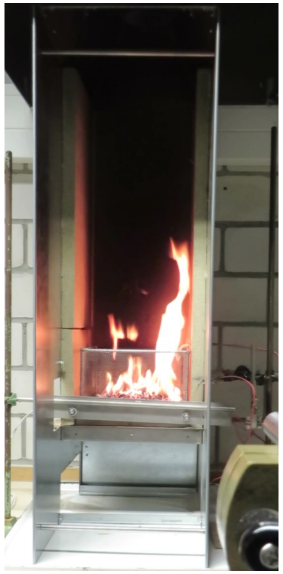	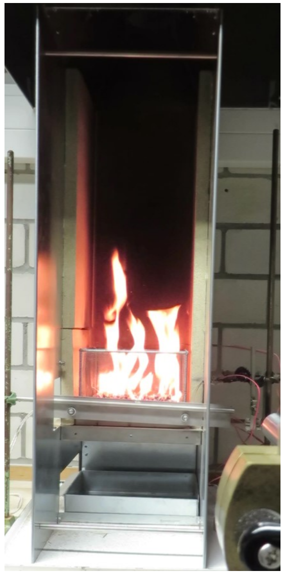
