# Supplementary material for: Do relationships between leaf traits and fire behaviour of leaf litter beds persist in time?
Source: PLoS One. 2018 Dec 26;13(12):e0209780. doi: 10.1371/journal.pone.0209780 (PMC6306239; doi:10.1371/journal.pone.0209780)

S6 Appendix. Details on measuring maximum flame height.

Videos were examined frame by frame with VideoPad video editor software (NCH Software, Greenwood Village, CO) and the frame with the highest flame was extracted. The dimensions of the combustion chamber were used to set scale in the ImageJ software. Height of the flame was always measured from the lower edge of the sand filled frame to the top of the flame plume (green arrow). This value was corrected for the height of the frame (2 cm). Furthermore, as flames start at the upper surface of the fuel, we also subtracted the average fuel bed depth of the given sample from the measured value. Thus, reported flame height for the picture below would be the length of the red arrow (frame + sample height) subtracted from the length of the green arrow.

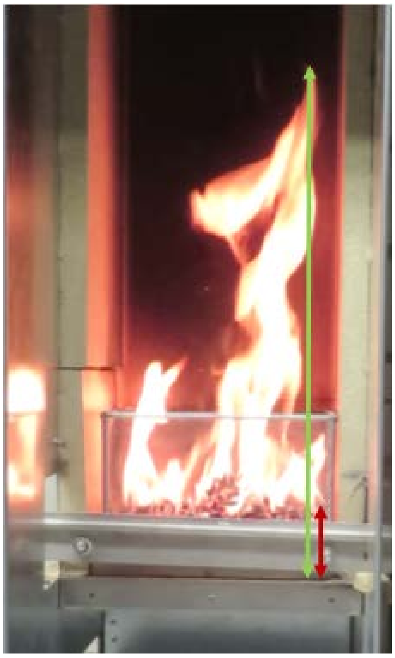

Supplement: S6 Appendix — (PDF) [file pone.0209780.s006.pdf]
