# Supplementary material for: Do relationships between leaf traits and fire behaviour of leaf litter beds persist in time?
Source: PLoS One. 2018 Dec 26;13(12):e0209780. doi: 10.1371/journal.pone.0209780 (PMC6306239; doi:10.1371/journal.pone.0209780)
Supplement: S7 Appendix — (PDF) [file pone.0209780.s007.pdf]

S7 Appendix. Relationships between packing ratio, bulk density and particle morphology.

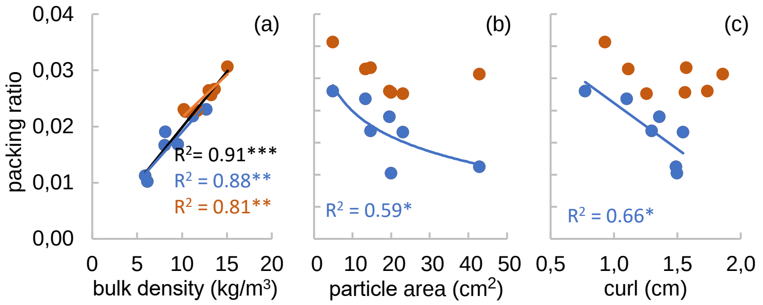

(a) bulk density (BD) and packing ratio, **(b)** particle area and packing ratio, **(c)** curl and packing ratio. The relationships were explored separately for fresh (blue points, lines and R^2 values) and settled (orange points, lines and R^2 values) samples, as well as on the whole data set (black lines and R^2 values). Regression line and R^2 values are indicated only for statistically significant relationships ($P \leq 0.05$.). $^*P \leq 0.05$, $^{**}P \leq 0.01$ and $^{***}P \leq 0.001$.
