# Supplementary material for: Do relationships between leaf traits and fire behaviour of leaf litter beds persist in time?
Source: PLoS One. 2018 Dec 26;13(12):e0209780. doi: 10.1371/journal.pone.0209780 (PMC6306239; doi:10.1371/journal.pone.0209780)
Supplement: S8 Appendix — (PDF) [file pone.0209780.s008.pdf]

S8 Appendix. Relationships between packing ratio and fire behaviour characteristics.

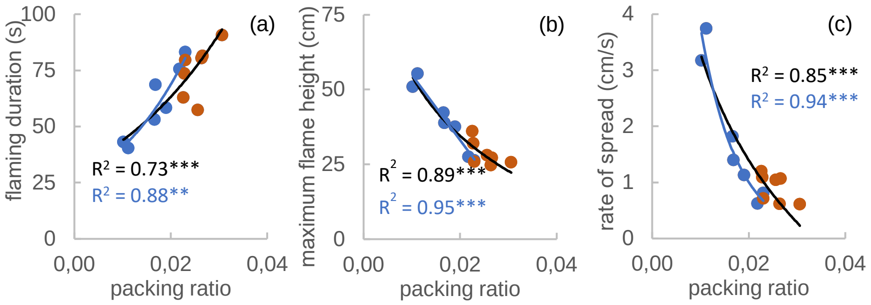

(a) packing ratio and flaming duration (FD), **(b)** packing ratio and maximum flame height (FH), **(c)** packing ratio and rate of spread (RoS). For each pair, relationships were explored separately for fresh (blue points, lines and R^2 values) and settled (orange points, lines and R^2 values) samples, as well as on the whole data set (black lines and R^2 values). Regression line and R^2 values are indicated only for statistically significant relationships ($P \leq 0.05$.). *$P \leq 0.05$, **$P \leq 0.01$ and ***$P \leq 0.001$.
