# Supplementary material for: Do relationships between leaf traits and fire behaviour of leaf litter beds persist in time?
Source: PLoS One. 2018 Dec 26;13(12):e0209780. doi: 10.1371/journal.pone.0209780 (PMC6306239; doi:10.1371/journal.pone.0209780)
Supplement: S9 Appendix — (PDF) [file pone.0209780.s009.pdf]

S9 Appendix. An example image of the flattened leaf litter particles.

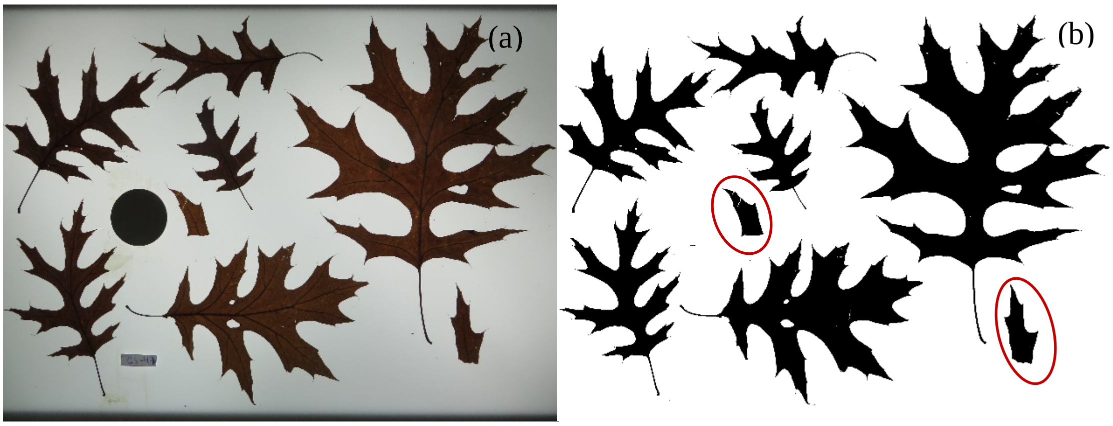

(a) Real colour image of a part of a spotted oak (*Quercus shumardii* Buckl.) subsample used for morphological measurements. The black circle was used as a reference object for scale calibration and had an area of 10 cm^2. The sample name is visible on each photo (written on a little paper square) to ensure sample traceability. **(b)** Binary, black and white, transformation of the image in (a). Objects other than leaves (edge, reference object, name tag) are removed. Two particles excluded from the area calculation are encircled in red. These were the only two particles, out of 15, excluded from the area calculation for this sample.
