# Supplementary material for: Do relationships between leaf traits and fire behaviour of leaf litter beds persist in time?
Source: PLoS One. 2018 Dec 26;13(12):e0209780. doi: 10.1371/journal.pone.0209780 (PMC6306239; doi:10.1371/journal.pone.0209780)
Supplement: S10 Appendix — (PDF) [file pone.0209780.s010.pdf]

## S10 Appendix. Sample mean values for area and curl.

| species | fresh (1) settled (2) | replicate | area* ($cm^2$) | curl (cm) |
|---|---|---|---|---|
| FE | 1 | 1 | 12,82 | 1,027 |
| FE | 1 | 2 | 11,69 | 1,273 |
| FE | 1 | 3 | 15,51 | 1,000 |
| FM | 1 | 1 | 22,78 | 1,413 |
| FM | 1 | 2 | 19,34 | 1,207 |
| FM | 1 | 3 | 16,60 | 1,447 |
| QI | 1 | 1 | 21,68 | 1,507 |
| QI | 1 | 2 | 25,72 | 1,347 |
| QI | 1 | 3 | 21,84 | 1,773 |
| QL | 1 | 1 | 13,02 | 0,993 |
| QL | 1 | 2 | 16,83 | 1,140 |
| QL | 1 | 3 | 14,26 | 1,760 |
| QP | 1 | 1 | 18,32 | 1,467 |
| QP | 1 | 2 | 22,12 | 1,547 |
| QP | 1 | 3 | 19,70 | 1,473 |
| QS | 1 | 1 | 36,69 | 1,087 |
| QS | 1 | 2 | 42,55 | 1,607 |
| QS | 1 | 3 | 49,40 | 1,767 |
| SD | 1 | 1 | 5,38 | 0,893 |
| SD | 1 | 2 | 4,94 | 0,600 |
| SD | 1 | 3 | 4,46 | 0,827 |
| FE | 2 | 1 | | 1,040 |
| FE | 2 | 2 | | 1,073 |
| FE | 2 | 3 | | 1,227 |
| FM | 2 | 1 | | 1,953 |
| FM | 2 | 2 | | 1,867 |
| FM | 2 | 3 | | 1,387 |
| QI | 2 | 1 | | 1,027 |
| QI | 2 | 2 | | 1,147 |
| QI | 2 | 3 | | 1,587 |
| QL | 2 | 1 | | 1,613 |
| QL | 2 | 2 | | 1,707 |
| QL | 2 | 3 | | 1,393 |
| QP | 2 | 1 | | 1,480 |
| QP | 2 | 2 | | 1,733 |
| QP | 2 | 3 | | 1,473 |
| QS | 2 | 1 | | 1,733 |
| QS | 2 | 2 | | 2,073 |
| QS | 2 | 3 | | 1,773 |
| SD | 2 | 1 | | 0,927 |
| SD | 2 | 2 | | 1,020 |
| SD | 2 | 3 | | 0,840 |

* area is given only for fresh treatment samples and represents area of the whole particles, as defined in the main article
