# Supplementary material for: Do relationships between leaf traits and fire behaviour of leaf litter beds persist in time?
Source: PLoS One. 2018 Dec 26;13(12):e0209780. doi: 10.1371/journal.pone.0209780 (PMC6306239; doi:10.1371/journal.pone.0209780)
Supplement: S11 Appendix — (PDF) [file pone.0209780.s011.pdf]

S11 Appendix. Measured values for fuel bed structure characteristics and fire behaviour characteristics referred to in the main article.

species	fresh (1) settled (2)	replicate	sample height (cm)	bulk density (kg/m^3)	ignition delay (s)	flaming duration (s)	flame height (cm)	rate of spread (cm/s)	sand temperature (°C)	smoldering duration (s)	unconsumed (%)	Testing order*	testing date	unique code+
FE	1	1	3,67	13,64	29,78	86,43	26,86	0,56	73,83	268,15	0,96%	11	13.12.2016	1FE
FE	1	2	4,17	12,00	34,84	76,41	35,18	0,59	85,6	150,10	0,96%	18	14.12.2016	2FE
FE	1	3	4,00	12,50	26,30	71,25	20,60	0,84	58,91	115,02	0,88%	22	15.12.2016	3FE
FE	1	4	4,50	11,11	20,95	80,70	26,47	0,48	55,1	239,75	1,24%	30	16.12.2016	4FE
FE	1	5	4,83	10,34	26,72	63,39	28,40	0,65	62,02	251,20	2,52%	41	19.12.2016	5FE
FM	1	1	6,00	8,33	28,20	54,82	35,63	1,25	53,78	202,29	0,99%	7	13.12.2016	1FM
FM	1	2	5,50	9,09	39,19	52,79	37,78	1,14	62,33	162,42	1,24%	20	14.12.2016	2FM
FM	1	3	5,33	9,38	27,96	53,35	38,12	1,36	67,72	201,44	2,52%	24	15.12.2016	3FM
FM	1	4	6,83	7,32	23,12	55,86	45,17	1,18	57,81	241,19	1,81%	33	16.12.2016	4FM
FM	1	5	5,67	8,82	16,42	74,88	30,89	0,72	62,84	158,75	1,05%	39	19.12.2016	5FM
QI	1	1	6,33	7,89	15,28	55,32	41,57	1,59	53,7	178,95	1,69%	9	13.12.2016	1QI
QI	1	2	5,67	8,82	23,85	44,07	44,42	2,56	71,41	141,69	0,60%	19	14.12.2016	2QI
QI	1	3	4,67	10,71	15,83	49,66	46,99	1,23	54,95	184,42	1,91%	25	15.12.2016	3QI
QI	1	4	6,17	8,11	25,63	50,81	38,33	2,12	57,39	234,33	1,20%	28	16.12.2016	4QI
QI	1	5	5,83	8,57	19,93	65,52	40,06	1,59	53,3	285,13	1,57%	38	19.12.2016	5QI
QL	1	1	5,50	9,09	27,92	62,85	46,86	1,33	55,38	351,82	2,57%	12	13.12.2016	1QL
QL	1	2	5,17	9,68	17,69	57,07	34,44	1,56	59,8	317,05	1,30%	16	14.12.2016	2QL
QL	1	3	5,50	9,09	48,83	58,06	36,12	1,45	63,12	379,15	0,94%	27	15.12.2016	3QL
QL	1	4	4,83	10,34	20,89	90,91	43,16	1,37	59,21	521,84	2,14%	32	16.12.2016	4QL
QL	1	5	4,33	11,54	26,72	74,48	33,53	1,28	61,53	434,86	1,55%	35	19.12.2016	5QL
QP	1	1	8,17	6,12	26,91	47,21	53,18	3,26	66,48	254,21	0,50%	10	13.12.2016	1QP
QP	1	2	7,83	6,38	75,22	38,88	53,87	2,45	62,67	370,86	0,59%	15	14.12.2016	2QP
QP	1	3	7,67	6,52	121,74	43,87	51,15	4,01	74,88	447,26	0,59%	23	15.12.2016	3QP
QP	1	4	8,00	6,25	67,46	38,21	58,41	3,07	59,24	279,68	0,49%	29	16.12.2016	4QP
QP	1	5	7,00	7,14	35,18	47,30	37,59	3,05	49,88	359,75	2,35%	36	19.12.2016	5QP
QS	1	1	7,50	6,67	61,13	38,61	44,16	2,49	54,96	432,62	3,83%	8	13.12.2016	1QS
QS	1	2	8,17	6,12	66,18	46,42	70,81	3,74	58,91	387,67	3,91%	17	14.12.2016	2QS
QS	1	3	8,17	6,12	106,29	40,36	49,16	4,05	47,51	429,48	3,32%	21	15.12.2016	3QS
QS	1	4	9,17	5,45	69,02	30,39	58,29	3,64	60,14	265,80	0,57%	34	16.12.2016	4QS
QS	1	5	7,50	6,67	51,22	46,41	54,03	4,80	55,62	255,85	1,56%	40	19.12.2016	5QS
SD	1	1	4,33	11,54	42,61	85,89	24,36	0,99	53,86	431,02	5,51%	13	13.12.2016	1SD
SD	1	2	3,17	15,79	40,94	97,04	26,14	0,62	66,73	536,37	4,23%	14	14.12.2016	2SD
SD	1	3	3,50	14,29	17,66	72,08	24,24	0,73	49,73	458,98	6,96%	26	15.12.2016	3SD
SD	1	4	5,00	10,00	45,32	81,10	32,25	0,93	63,81	484,62	1,46%	31	16.12.2016	4SD
SD	1	5	3,17	15,79	29,32	80,04	24,74	0,78	53,84	475,14	8,31%	37	19.12.2016	5SD

species	fresh (1) settled (2)	replicate	sample height (cm)	bulk density (kg/m^3)	ignition delay (s)	flaming duration (s)	flame height (cm)	rate of spread (cm/s)	sand temperature (°C)	smoldering duration (s)	unconsumed (%)	Testing order*	testing date	unique code+
FE	2	1	3,50	14,29	14,56	89,22	22,67	0,55	55,86	619,66	4,12%	5	22.02.2017	33FE-5
FE	2	2	3,00	16,67	20,34	76,83	30,68	0,68	45,76	307,38	2,48%	8	23.02.2017	15FE-3
FE	2	3	2,67	18,75	32,14	78,42	27,34	0,68	49,21	196,90	3,61%	16	23.02.2017	37FE-6
FE	2	4	4,17	12,00	29,99	86,26	20,60	0,50	42,76	192,46	3,29%	25	27.02.2017	4FE-1
FE	2	5	3,00	16,67	41,66	71,89	22,29	0,66	47,24	225,87	4,51%	32	28.02.2017	46FE-7
FM	2	1	4,00	12,50	14,12	82,41	15,36	0,54	56,03	283,85	4,15%	2	22.02.2017	30FM-5
FM	2	2	4,67	10,71	22,06	79,24	30,05	0,77	49,93	240,24	5,03%	14	23.02.2017	21FM-3
FM	2	3	4,17	12,00	33,77	78,27	24,27	0,69	51,11	152,05	2,18%	20	24.02.2017	41FM-6
FM	2	4	4,67	10,71	17,63	79,94	23,68	0,82	62,91	180,85	2,51%	22	27.02.2017	1FM-1
FM	2	5	5,17	9,68	35,39	78,06	36,19	0,75	51,88	260,16	2,17%	33	28.02.2017	47FM-7
QI	2	1	4,33	11,54	21,32	51,22	36,94	1,07	53,44	353,93	2,43%	3	22.02.2017	31QI-5
QI	2	2	4,83	10,34	67,12	50,55	40,98	1,95	58,74	209,42	1,26%	9	23.02.2017	16QI-3
QI	2	3	4,17	12,00	20,23	59,33	35,44	1,31	58,88	139,26	0,30%	15	23.02.2017	36QI-6
QI	2	4	5,50	9,09	11,11	71,82	33,59	0,84	56,12	252,39	1,09%	27	27.02.2017	6QI-1
QI	2	5	4,33	11,54	15,03	81,84	33,27	0,83	62,25	335,30	1,84%	34	28.02.2017	48QI-7
QL	2	1	3,17	15,79	16,39	81,93	28,67	0,78	46,5	471,47	5,96%	6	22.02.2017	34QL-5
QL	2	2	3,83	13,04	37,32	86,90	24,95	1,32	52,14	572,80	2,45%	11	23.02.2017	18QL-3
QL	2	3	3,33	15,00	12,01	86,85	24,93	0,88	58,7	382,33	2,66%	18	24.02.2017	39QL-6
QL	2	4	4,00	12,50	35,81	84,18	34,13	1,37	52,47	520,60	3,52%	23	27.02.2017	2QL-1
QL	2	5	2,83	17,65	51,60	67,56	23,07	0,99	47,36	375,78	13,82%	31	28.02.2017	45QL-7
QP	2	1	3,83	13,04	15,19	90,21	34,47	0,94	36,28	338,35	14,56%	1	22.02.2017	29QP-5
QP	2	2	4,50	11,11	25,07	54,86	36,07	1,40	37,08	343,45	22,90%	12	23.02.2017	19QP-3
QP	2	3	3,50	14,29	20,27	100,49	38,03	0,88	58,05	327,55	2,85%	21	24.02.2017	42QP-6
QP	2	4	4,17	12,00	15,98	53,77	27,76	1,01	45,29	317,75	30,05%	28	27.02.2017	7QP-1
QP	2	5	4,17	12,00	12,26	69,22	23,39	1,23	57,87	366,99	6,59%	30	28.02.2017	44QP-7
QS	2	1	3,00	16,67	12,81	52,81	22,73	0,71	30,25	483,81	39,15%	7	22.02.2017	35QS-5
QS	2	2	3,33	15,00	20,26	53,60	29,38	1,08	34,29	400,87	28,94%	10	23.02.2017	17QS-3
QS	2	3	4,33	11,54	10,20	52,12	29,08	1,03	37,44	259,88	29,55%	19	24.02.2017	40QS-6
QS	2	4	3,33	15,00	31,74	51,41	34,71	1,60	28,77	262,96	37,49%	24	27.02.2017	3QS-1
QS	2	5	4,00	12,50	17,54	77,38	24,26	0,81	23,88	202,65	42,26%	29	28.02.2017	43QS-7
SD	2	1	2,83	17,65	45,73	86,91	26,65	0,56	49,8	604,36	12,28%	4	22.02.2017	32SD-5
SD	2	2	3,00	16,67	28,71	92,22	24,48	0,72	54,28	282,03	6,29%	13	23.02.2017	20SD-3
SD	2	3	3,17	15,79	35,39	98,88	24,31	0,58	53,64	339,00	9,88%	17	23.02.2017	38SD-6
SD	2	4	3,33	15,00	16,61	95,90	26,93	0,64	53,28	440,28	6,36%	26	27.02.2017	5SD-1
SD	2	5	3,00	16,67	23,78	79,64	25,70	0,54	45,05	292,11	3,87%	35	28.02.2017	49SD-7

* Testing order refers to order in which samples were tested within the treatment. Ordering of samples within the fresh treatment starts with number 7. First 6 tests were used for perfecting the testing procedure. Surplus samples were used for this purposes.

\+ Upon weighting samples were randomly attributed to a treatment and each sample received an unique code. All the data and records (e.g. video records, temperature records, top and side photos of the settled samples…) refering to that specific sample contain this code

Species are indicated as follows: FE = European ash (*Fraxinus excelsior* L.), FM = Manchurian ash (*Fraxinus mandshurica* Rupr.), QI = shingle oak (*Quercus imbicaria* Michx.), QL = Lebanon oak (*Quercus libani* Olivier), QP = pin oak (*Quercus palustris* Münchh.), QS = spotted oak (*Quercus shumardii* Buckl.) and SD = service tree (*Sorbus domestica* L.).
