# Supplementary material for: Do relationships between leaf traits and fire behaviour of leaf litter beds persist in time?
Source: PLoS One. 2018 Dec 26;13(12):e0209780. doi: 10.1371/journal.pone.0209780 (PMC6306239; doi:10.1371/journal.pone.0209780)
Supplement: S12 Appendix — (PDF) [file pone.0209780.s012.pdf]

S12 Appendix. Effects of the settling treatment on some additional characteristics.

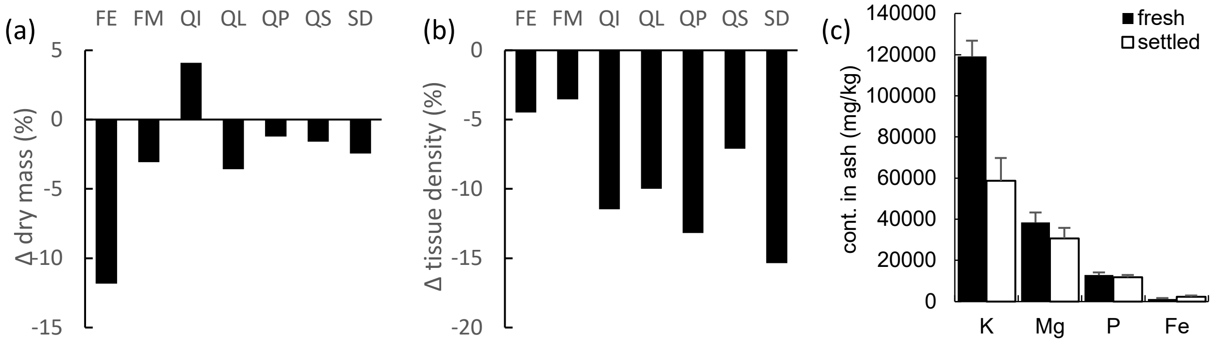

(a) Percent change of the species dry mass after the settling treatment, **(b)** percent change of the species tissue density after the settling treatment, **(c)** content (average and standard error) of potassium (K), magnesium (Mg), phosphorous (P) and iron (Fe) in the *F. excelsior* ash of the fresh and settled treatment. Species are indicated as follows: *F. excelsior* (FE), *F. mandshurica* (FM), *Q. imbicaria* (QI), *Q. libani* (QL), *Q. palustris* (QP), *Q. shumardii* (QS), *S. domestica* (SD). Mineral content was determined by inductively coupled plasma atomic emission spectroscopy (ICP-AES) at the Analytical Chemistry Unit, Core Facility Hohenheim. Analyses were conducted on three randomly chosen ash samples for each treatment. Each sample was measured twice and the average value of two measurements was taken for further calculations (i.e. standard error is based on sample average, N = 3). Results are presented for *F. excelsior*, as only for this species all the samples were measured twice. Besides leaching of minerals (K, Mg, P), upwards transport of minerals from the soil into the sample (e.g. for iron Fe) can be observed.
